# Operation of a TCA cycle subnetwork in the mammalian nucleus

**DOI:** 10.1126/sciadv.abq5206

**Published:** 2022-08-31

**Authors:** Eleni Kafkia, Amparo Andres-Pons, Kerstin Ganter, Markus Seiler, Tom S. Smith, Anna Andrejeva, Paula Jouhten, Filipa Pereira, Catarina Franco, Anna Kuroshchenkova, Sergio Leone, Ritwick Sawarkar, Rebecca Boston, James Thaventhiran, Judith B. Zaugg, Kathryn S. Lilley, Christophe Lancrin, Martin Beck, Kiran Raosaheb Patil

**Affiliations:** ^1^ European Molecular Biology Laboratory (EMBL), Heidelberg, Germany.; ^2^ The Medical Research Council Toxicology Unit, University of Cambridge, Cambridge, UK.; ^3^ Friedrich Miescher Institute for Biomedical Research, Basel, Switzerland.; ^4^ European Molecular Biology Laboratory (EMBL), Rome, Italy.; ^5^ Buchmann Institute for Molecular Life Sciences, Goethe University Frankfurt, Frankfurt, Germany.; ^6^ Department of Biochemistry, University of Cambridge, Cambridge, UK.; ^7^ VTT Technical Research Center of Finland, Helsinki, Finland.; ^8^ Max Planck Institute of Biophysics, Frankfurt, Germany.

## Abstract

Nucleic acid and histone modifications critically depend on the tricarboxylic acid (TCA) cycle for substrates and cofactors. Although a few TCA cycle enzymes have been reported in the nucleus, the corresponding pathways are considered to operate in mitochondria. Here, we show that a part of the TCA cycle is operational also in the nucleus. Using ^13^C-tracer analysis, we identified activity of glutamine-to-fumarate, citrate-to-succinate, and glutamine-to-aspartate routes in the nuclei of *HeLa* cells. Proximity labeling mass spectrometry revealed a spatial vicinity of the involved enzymes with core nuclear proteins. We further show nuclear localization of aconitase 2 and 2-oxoglutarate dehydrogenase in mouse embryonic stem cells. Nuclear localization of the latter enzyme, which produces succinyl-CoA, changed from pluripotency to a differentiated state with accompanying changes in the nuclear protein succinylation. Together, our results demonstrate operation of an extended metabolic pathway in the nucleus, warranting a revision of the canonical view on metabolic compartmentalization.

## Teaser

^13^C-tracer analysis reveals glutamine-to-fumarate, citrate-to-succinate, and glutamine-to-aspartate pathways in the nucleus.

## INTRODUCTION

Several of the chemical moieties and cofactors required for the covalent modifications of chromatin and RNA—acetyl–CoA (coenzyme A), α-ketoglutarate, succinyl-CoA, 2-hydroxyglutarate, succinate, and fumarate—originate in the tricarboxylic acid (TCA) cycle (*1*–*9*). Thus, beyond its canonical biosynthetic and bioenergetic role in cell function, the TCA cycle also plays a fundamental role in the spatiotemporal regulation of gene expression as well as in genome repair (*5*, *6*, *10*–*17*). The metabolites required for these modifications, or their direct precursors, are generally assumed to diffuse from mitochondria to the nuclear sites of need. This diffusion-centric scenario, however, contrasts with the emerging understanding of intracellular molecular crowding and phase separation (*18*) and disregards the reaction-diffusion case wherein the metabolite of interest can be en route sequestered by other enzymes. These considerations raise the possibility that some of the metabolites are produced inside the nucleus to ensure a timely supply to the corresponding nuclear processes. In support of this hypothesis, three individual TCA cycle enzymes [pyruvate dehydrogenase complex (PDC); α-ketoglutarate dehydrogenase complex (OGDC), and fumarate hydratase (FH)] have been reported to be present in the nucleus (*14*, *19*–*23*). However, these observations only partially alleviate the concerns against the diffusion-centric model. In particular, whether the precursors of these reactions, some of which are not highly abundant or stable in the cell, are diffusing into the nucleus or are, in turn, produced by other reactions occurring in the nucleus is unclear. A nuclear pyruvate–to–α-ketoglutarate route was recently described, albeit during a specific stage of mouse embryonic development (*24*). Considering the problems with the diffusion-centric model and the critical importance of multiple TCA cycle intermediates in chromatin and RNA modifications, we hypothesized that a large part of this metabolic network is operational in the mammalian nucleus.

## RESULTS

**Fig. 1.**
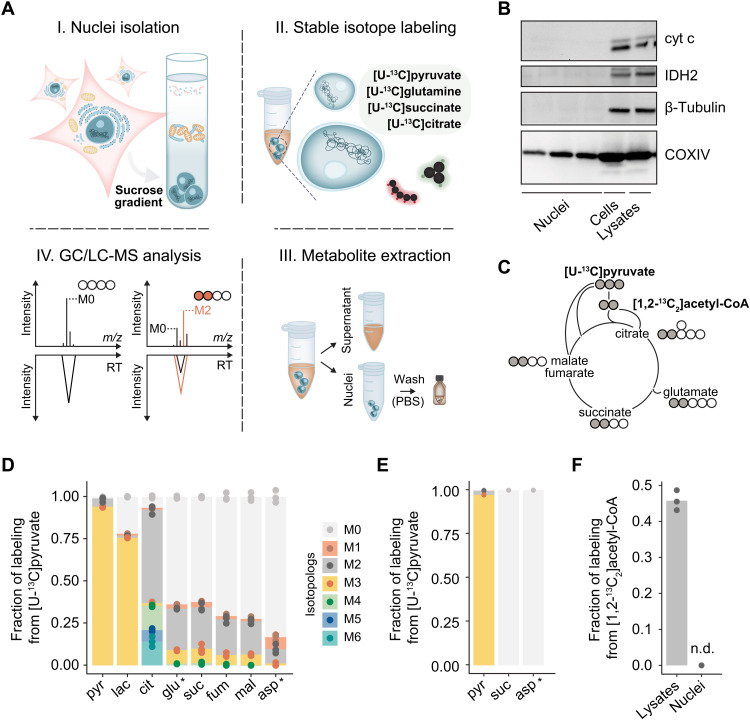
Probing the functional presence of multistep metabolic pathways in the nucleus. (**A**) Schematic overview of the ^13^C-labeling strategy in isolated nuclei. (**B**) Immunoblot analysis of isolated nuclei, cells, and whole-cell lysates for the mitochondrial shuttling protein cytochrome c (cyt c), the mitochondrial matrix protein IDH2, the cytoplasmic protein β-tubulin, and the inner mitochondrial membrane protein COXIV. For the isolated nuclei, the data shown are from three independent nuclear isolations. (**C**) Diagram showing the expected main mass isotopolog transitions of the TCA cycle intermediates in whole-cell lysates incubated with [U-^13^C]pyruvate. Gray and empty circles represent ^13^C and ^12^C carbons, respectively. (**D** and **E**) Fraction of labeling of the different mass isotopologs (Mn; *n*, number of ^13^C carbons) for each metabolite in whole-cell lysates (D) and nuclei (E) incubated with [U-^13^C]pyruvate. Note that for glutamate (glu*) and aspartate (asp*), the quantified ions correspond to a four- and a three-carbon fragment, respectively. (**F**) ^13^C labeling for the M2 isotopolog of citrate in whole-cell lysates and in nuclei incubated with [1,2-^13^C_2_]acetyl-CoA. In (D) to (F), whole-cell lysates and nuclei correspond to three biological replicates (*n* = 3). For (D) to (F), data are presented as the mean of the indicated biological replicates with individual data points shown. pyr, pyruvate; lac, lactate; cit, citrate; glu, glutamate; suc, succinate; fum, fumarate; mal, malate; asp, aspartate; n.d., not detected.

To investigate the metabolic pathways operating in the nucleus, we incubated isolated nuclei from *HeLa* cells with uniformly ^13^C-labeled substrates representing key entry points or constituents of the TCA cycle (see Fig. 1A and Materials and Methods). The nuclei were then washed to remove any extranuclear material to capture only the nucleoplasmic metabolite pools. In parallel, lysed cells were used as a control reflecting the enzymatic activities at the whole-cell level. The metabolic extracts were analyzed with gas and liquid chromatography coupled to mass spectrometry (GC-MS and LC-MS, respectively) to quantify the relative abundances of the mass isotopologs of the TCA cycle intermediates.

To assess the purity of the nuclei isolations, we tested for the presence of the cytoplasmic protein β-tubulin and two mitochondria-specific proteins, isocitrate dehydrogenase 2 (IDH2) and cytochrome c, representing mitochondrial matrix and transmembrane shuttling activity, respectively. All three were not detected in the nuclear preparations (Fig. 1B). While the isolated nuclei appeared to carry fragments of mitochondrial membranes [cytochrome c oxidase subunit 4 isoform 1 (COXIV); Fig. 1B], we verified by electron microscopy that the preparations were free of whole mitochondria (fig. S1). To quantitatively assay whether the mitochondrial membrane fragments interfered with the metabolite labeling, we contrasted the metabolic activities of the nuclei isolations and lysed cells following incubation with [U-^13^C]pyruvate. As expected, lysed cells were labeled in all measured TCA cycle metabolites (Fig. 1, C and D). Conversely, in isolated nuclei, the two detected TCA cycle intermediates, succinate and aspartate, did not incorporate any ^13^C in their carbon backbones (Fig. 1E). Similarly, supplementation of nuclei with [1,2-^13^C_2_]acetyl-CoA did not result in ^13^C enrichment, whereas lysed cells readily used [1,2-^13^C_2_]acetyl-CoA to synthesize citrate (M2 isotopolog; Fig. 1F). These results were consistent with previous studies in isolated nuclei from mammalian cells where pyruvate carbon entry in TCA metabolites other than acetyl-CoA was not observed (*19*, *25*). Collectively, these control experiments mark the purity of the nuclei isolations regarding their enzymatic content.

**Fig. 2.**
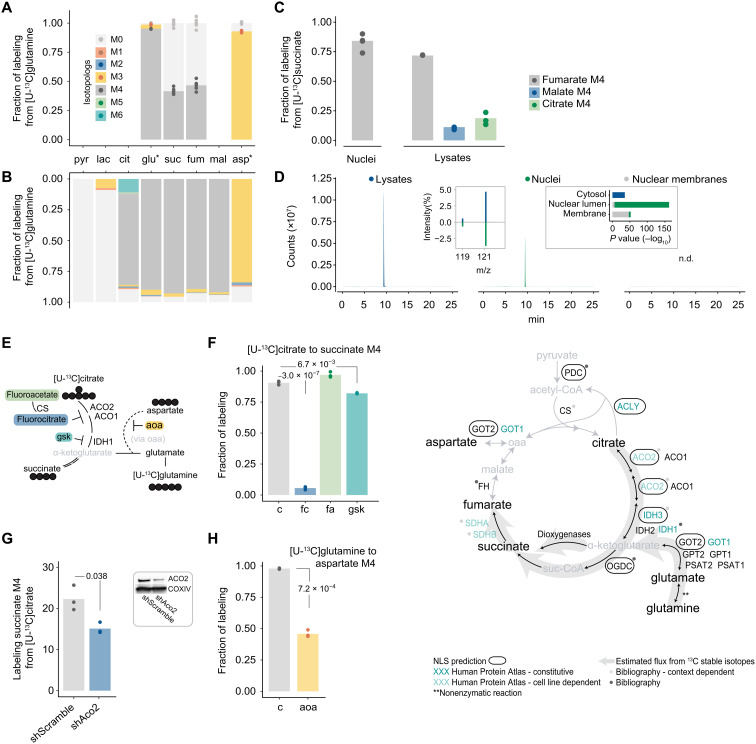
Glutamine and citrate feed a TCA cycle subnetwork in the nucleus. (**A** and **B**) ^13^C labeling observed as mass isotopologs (Mn; *n*, number of ^13^C carbons) in nuclei (A) and in whole-cell lysates (B) incubated with [U-^13^C]glutamine. glu* and asp* denote four- and three-carbon fragments, respectively. (**C**) ^13^C labeling for the M4 isotopolog of fumarate, malate, and citrate in nuclei and whole-cell lysates incubated with [U-^13^C]succinate. (**D**) Representative ion chromatogram and mass spectrum (left inset) of the M4 isotopolog of succinate (*m/z* 121.0322) in samples incubated with [U-^13^C]citrate. Results of GO enrichment (cellular component) in proteomic measurements from whole-cell lysates and nuclear preparations shown in the right inset. (**E**) Potential enzymatic steps and corresponding inhibitors for the identified nuclear TCA cycle subnetwork. (**F**) ^13^C labeling for the succinate M4 isotopolog in nuclei incubated with [U-^13^C]citrate (c) or [U-^13^C]citrate combined with fluorocitrate (fc), fluoroacetate (fa), or gsk864 (gsk). (**G**) Effect of shRNA knockdown of aconitase 2 (sh*Aco2*) on the succinate M4 isotopolog in nuclei incubated with [U-^13^C]citrate. shScramble, nontargeting shRNA control. (**H**) Effect of AOA (aoa) on the aspartate M4 isotopolog in nuclei incubated with [U-^13^C]glutamine. (**I**) Schematic of the TCA cycle overlaid with the identified nuclear flux routes, NLS predictions, and other data sources. At least three biological replicates were used in all experiments.

In mitochondria, glutamine/glutamate is one of the main anaplerotic entry points feeding the TCA cycle, predominantly through α-ketoglutarate (*26*, *27*). Considering that glutamine is among the most abundant amino acids in several tissues, in blood, and in common culture media (*28*), it is unlikely to be limited because of diffusion constraints. We therefore investigated whether it could supply the nucleus with the downstream metabolic intermediates of the TCA cycle. Tracing [U-^13^C]glutamine in isolated *HeLa* nuclei, we observed ^13^C enrichment in glutamate, succinate, fumarate, and aspartate (Fig. 2A). While the fractional labeling of glutamate and aspartate reached 90% already after 1 hour of incubation (fig. S2A), succinate and fumarate ^13^C fractions reached circa 45% after 5 hours (Fig. 2A and fig. S2A). The ^13^C fraction of succinate at 5 hours was smaller than that at 1 hour, consistent with its conversion to fumarate, the labeling of which showed a proportionate increase from 1 to 5 hours (fig. S2A). The control experiment with the lysed cells revealed additional metabolites being ^13^C labeled, including malate and citrate, illustrating the activity of the whole TCA cycle as expected (Fig. 2B). To rule out the possibility that the absence of ^13^C-enriched intermediates downstream of fumarate in the nucleus was due to substrate concentration limitations, we incubated the nuclei (and lysed cells as a control) with [U-^13^C]succinate. While the lysed cells again showed the incorporation of ^13^C in fumarate, malate, and citrate, in the nuclei only ^13^C fumarate was detected (Fig. 2C), in agreement with the [U-^13^C]glutamine tracer experiment.

To assess the utilization of carbon sources other than glutamine in the nucleus, we next focused on citrate, a key metabolic intermediate known to locally provide acetyl-CoA for chromatin modifications (*15*, *29*). To confirm that enzyme-mediated biosynthesis is not limited by the buffer composition (e.g., by the cofactors), we first validated that *HeLa* nuclei could use [U-^13^C]citrate as a substrate to synthesize acetyl-CoA (M2 isotopolog; table S4). Further to acetyl-CoA, the supplementation of nuclei with [U-^13^C]citrate resulted in the ^13^C labeling of succinate (Fig. 2D). The absence of labeled fumarate in nuclei and in the lysed cells (fig. S2B) was likely due to the known properties of citrate as a metal chelator and allosteric inhibitor of several metabolic enzymes (*30*). Next, we isolated and incubated nuclear membranes (table S14) with [U-^13^C]citrate. Following 5 hours of incubation, we did not detect any ^13^C enrichment apart from the [U-^13^C]citrate substrate itself (Fig. 2D and table S6), further supporting the intranuclear topology of the citrate-to-succinate axis.

To begin to ascertain the enzymatic nature of the glutamine/glutamate– and citrate-to-succinate conversions, we next used small-molecule inhibitors for relevant enzymatic steps in the presence of the respective ^13^C substrates (Fig. 2E). Fluorocitrate, a competitive inhibitor of aconitase (ACO1 and ACO2) (*31*) that catalyzes the first reaction in the citrate-to-succinate route, led to a significant reduction in the ^13^C fractional labeling of succinate following incubation with [U-^13^C]citrate (Fig. 2F), corroborating that aconitase is active in the *HeLa* nucleus. Conversely, the presence of fluoroacetate did not alter the ^13^C succinate enrichment (Fig. 2F) since its inhibitory effect requires first its enzymatic conversion to fluoroacetyl-CoA and fluorocitrate, including the activity of citrate synthase (CS) (Fig. 2E) (*31*). Downstream of aconitase, the next enzymatic step is mediated by isocitrate dehydrogenase. GSK864, an allosteric inhibitor of isocitrate dehydrogenase 1 (IDH1) (*32*), significantly, albeit partially, reduced the ^13^C labeling of succinate (Fig. 2F). The partial reduction is not unexpected as this inhibitor is targeted at the mutant versions of IDH1 and IDH3 (*33*). The small-molecule inhibitor results were further confirmed through short hairpin RNA (shRNA)–mediated knockdown of ACO2 (Fig. 2G), which decreased the conversion of citrate to succinate. Last, in the presence of [U-^13^C]glutamine, supplementation of isolated nuclei with aminooxyacetate (AOA), an inhibitor of the pyridoxal-5′-phosphate–dependent transaminases (*26*, *34*), resulted in a marked decrease in the ^13^C labeling of aspartate (Fig. 2H), indicating that transamination-driven α-ketoglutarate synthesis from glutamate is the main route.

The results from the glutamine and citrate labeling experiments support these as sources of TCA cycle intermediates in the *HeLa* nucleus. To examine the nuclear presence of the corresponding enzymes more broadly, we next explored the Human Protein Atlas images (*35*). We observed that ACO2 and IDH3 that catalyze the citrate–to–α-ketoglutarate reactions exhibit nuclear localization that is either cell line dependent (ACO2) or ubiquitous (IDH3G) across all examined cells (Fig. 2I). Additional TCA cycle enzymes, as well as enzymes that catalyze equivalent reactions elsewhere in the cell, likewise were found to exhibit a signal for nuclear localization. These included IDH1, succinate dehydrogenase subunits A (SDHA) and B (SDHB), and various aminotransferases (e.g., GOT1) responsible for glutamate carbon entry to TCA cycle through α-ketoglutarate formation (Fig. 2I). Mutant IDH1 and the heterodimer SDHA-SDHB have been previously reported in the nucleus, albeit with no functional relevance (*36*, *37*). Furthermore, published proteomic data from isolated nuclei included several TCA cycle components (*38*). We additionally confirmed the presence of TCA enzymes in isolated *HeLa* nuclei quantitatively compared to lysed cells and isolated nuclear membranes by proteomic analysis (table S14). We therefore searched the sequences of TCA cycle enzyme for nuclear localization signals (NLS). We found putative canonical NLS for every enzymatic step, save CS, from pyruvate up to the generation of succinyl-CoA (Fig. 2I). Collectively, these independent and orthogonal evidence—Human Protein Atlas, proteomics, and NLS—provide a localization support to every enzymatic step implicated by our functional labeling results.

To further attest the nuclear localization of the implicated metabolic route, we next aimed to identify interacting and proximal proteins for a subset of TCA cycle enzymes using in vivo proximity-dependent biotinylation (BioID) coupled to MS (*39*). The selected bait enzymes covered part of the citrate-to-succinate axis: ACO2, IDH3G subunit from IDH3 complex, IDH1, and OGDH subunit from OGDC complex. As a negative control, we used IDH2, a mitochondrial enzyme with no evidence for nuclear localization. We also included, despite having no evidence of downstream TCA cycle products in our labeling assays, pyruvate dehydrogenase B (PDHB) as a potential nuclear localized enzyme based on previous reports (*19*, *21*).

**Fig. 3.**
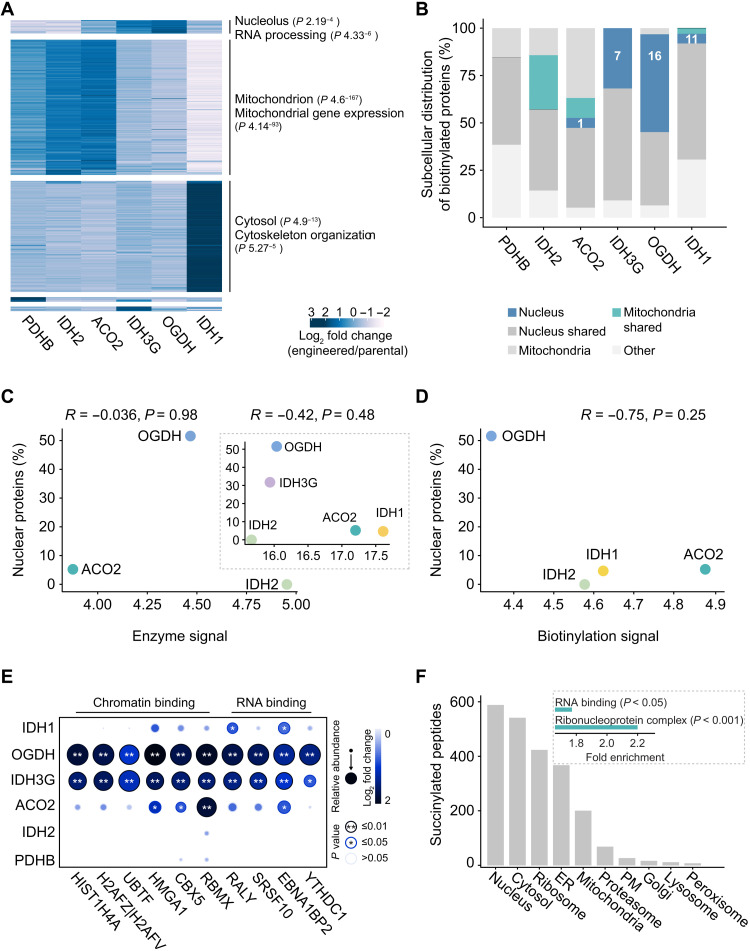
Proximity biotinylation MS reveals core nuclear proteins as putative interactors of IDH1, IDH3G, OGDH, and ACO2. (**A**) Overview of the identified interactors of each bait enzyme. Heatmap shows fold changes in the abundance of each putative interactor in cells expressing the bait enzyme compared to the parental cells. Top: Significantly enriched GO terms for cellular component (top) and “biological process” (bottom) are shown for each cluster. (**B**) Subcellular distribution of the putative interactors of each bait enzyme. Nucleus and mitochondria refer to proteins detected only in the nucleus and mitochondria, respectively. Nucleus shared, proteins detected in the nucleus and any other compartment. Mitochondria shared, proteins detected in the mitochondria and any other compartment excluding nucleus. (**C**) Correlation between the percentage of interacting nuclear proteins and the abundance of the bait enzymes; the latter was estimated using immunofluorescence (main panel) or proteomics (inset). (**D**) Correlation between the percentage of interacting nuclear proteins and the biotinylation levels in cells expressing the bait enzymes. (**E**) Selected putative interactors of the bait enzymes. (**F**) Subcellular distribution of succinylated peptides in U2OS cells quantified by LOPIT. Inset: Enriched GO terms for nuclear proteins with succinylated peptides. ER, endoplasmic reticulum; PM, plasma membrane.

Gene ontology (GO) enrichment analysis of the results from the BioID assay for these five enzymes affirmed that the vast majority of the biotinylated proteins (hereafter termed putative interactors) resided in the primary location of the corresponding bait enzyme—the mitochondria for all, except IDH1, which mainly localizes to the cytoplasm (Fig. 3A, clusters 2 and 3, and tables S7 and S8). However, we identified a group of putative interactors, shared by OGDH and IDH3G, featuring the nucleolus as the top enriched subcellular compartment (Fig. 3A, cluster 1, and tables S7 and S8). This cluster did not show any interactions with PDHB and IDH2, consistent with the sole mitochondrial localization of the latter (tables S3 and S4). GO analysis also highlighted activities closely linked with the nucleolus, notably RNA binding and metabolic processing of ribosomal RNAs (Fig. 3A and table S8). This is in agreement with recent studies reporting TCA cycle enzymes as RNA binding proteins (*40*, *41*). Together, these data, while attesting the localization of the bait enzymes to their primary compartment, also support their additional nuclear presence, particularly discernible for OGDH and IDH3G.

To assess the localization of the interactors of the TCA cycle enzymes more stringently, we next identified the top putative interactors for each of the bait enzymes by contrasting against IDH2 hits (negative control, mitochondrial) (Materials and Methods). Inspection of the known localizations of the resulting putative interactors revealed a fraction characterized as exclusively nuclear for OGDH, IDH3G, IDH1, and ACO2 (Fig. 3B and table S9). No nuclei-only localized putative interactors were detected for the mitochondria-exclusive IDH2 (as compared against ACO2). None of the PDHB interactors also show exclusive nuclear localization, despite its reported nuclear presence, which can be attributed to the cell cycle phase–dependent localization to the nucleus (*19*). We also checked whether the capture of nuclear proteins could be attributed to the high abundance of the bait proteins suggestive of noise or artifacts. No such bias was observed, regardless of whether the abundances were estimated by using immunofluorescence, MS (*42*) (Fig. 3C and fig. S3A), or the whole-cell biotinylation levels (Fig. 3D and fig. S3B). Among the putative nuclear interaction partners, in addition to RNA binding proteins, we identified chromatin binding and remodeling factors (e.g., RBMX and HMGA1) and specific histones (e.g., HIST1H4A and H2AFZ) (Fig. 3E and table S9). The latter is in accord with the recently reported interaction between OGDC and acetyltransferase 2A mediating the succinylation of histone lysine residues (*22*).

Succinylation has been previously observed for many nuclear proteins (*43*). However, aside from the specific case of succinylation of histone proteins (*22*), succinylation of nuclear proteins has been detected in whole-cell lysates without information about their localization. The charge and modification state of lysine residues may affect the RNA interactions in canonical RNA binding or protein localizations (*44*). Succinylation inverts the charge of lysine residues and adds a bulky adduct, therefore imposing a significant functional impact (*45*, *46*) on the proteins. However, the origin of the required substrate succinyl-CoA in the nucleus is yet unclear as no mitochondrial transport mechanism is known. To test whether the presence of OGDH in the nucleus that we observed contributes to nuclear succinylation, we used the localization of organelle proteins by isotope tagging (LOPIT) methodology (*47*) with additionally including a succinyl-peptide enrichment step in U2OS cells. This revealed the nucleus as the compartment with the highest number of succinylated peptides (Fig. 3F). Succinylation was more readily detected in the most abundant proteins (fig. S8C). Taking this bias into account, GO enrichment analysis for the nuclear succinylated proteins annotated as significantly overrepresented the terms “ribonucleoprotein complex” (2.2-fold enriched, adjusted *P* < 0.001) and “RNA binding” (1.7-fold enriched, adjusted *P* < 0.05) (Fig. 3F). These results are in accordance with the enrichment of RNA binding proteins in the putative interactors and proximal proteins of OGDH (Fig. 3E and table S9), suggesting that synthesis of succinyl-CoA leads to succinylation of OGDH’s nuclear neighborhood. Together, the succinylation analysis supports the hypothesis that the presence of TCA cycle enzymes in the nucleus is linked to the supply of epigenetic modification factors.

**Fig. 4.**
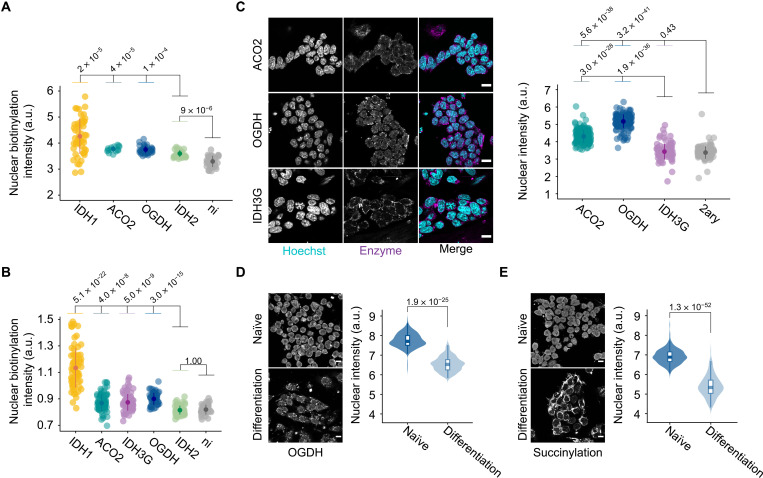
The subcellular distribution of metabolic enzymes and their putative interactors reveals their nuclear neighborhood. (**A** and **B**) Putative interactor signal (biotinylation signal measured using fluorescently labeled streptavidin) in the nuclear region of whole cells (A) or isolated nuclei (B) expressing the bait enzymes. “ni” refers to cells where the expression of the bait enzymes was not induced. (**C**) Representative immunofluorescence images and quantification of ACO2, OGDH, and IDH3G in the nucleus of mouse embryonic stem cells. The quantification was based on immunofluorescence using antibodies against the endogenous enzymes. “2ary” refers to cells stained only with the secondary, fluorophore-conjugated, antibody. DNA was stained with Hoechst (cyan). Scale bars, 15 μm. (**D** and **E**) Representative immunofluorescence images and quantification of OGDH (D) and succinylation (E) in naïve mouse embryonic stem cells and in differentiated counterparts. Scale bars, 10 μm. In (A) to (E), points represent quantification (log_2_ scale) of individual cells or nuclei, with the population mean and SD calculated for each case. In (B), (D), and (E), the biotinylation, OGDH, and succinylation levels were normalized to Hoechst signal. In (A) to (E), significance was assessed using the Wilcoxon rank sum test. a.u., arbitrary units.

We next visualize the subcellular distribution of selected bait enzymes and their corresponding putative interactors using immunofluorescence microscopy. The enzymes were detected in their expected primary compartment, while IDH3G was additionally present in the region of the nucleolus (fig. S4). The staining pattern of the putative interactors (corresponding to the biotinylation signal as assessed with fluorescently labeled streptavidin) followed the primary localization of the respective bait enzymes, while a discernible nuclear signal was detected only in the case of IDH1 (Fig. 4A and fig. S5). To minimize the more abundant mitochondrial staining that could obscure the nuclear signal in all other cases for which the nuclear localization was suggested by our labeling and BioID assays (i.e., OGDH, IDH3G, and ACO2), we examined the staining for the putative interacting proteins (biotinylation) in isolated nuclei. For the case of IDH2, the nuclear biotinylation levels were similar to those of cells not expressing a bait enzyme (Fig. 4B and fig. S6, B and C), attesting the sole mitochondrial location. In comparison, in all other cases, there was a significantly higher degree of nuclear biotinylation (*P* < 0.01) (Fig. 4B and fig. S6, B and C). Together with the BioID MS results, the microscopically visible putative interacting partners in the region of the nucleus for IDH1, ACO2, OGDH, and IDH3G further support their nuclear presence.

The visibility of the putative interactors in isolated nuclei but not in whole cells is in line with the expected low abundance of the enzymes in the nucleus relative to their primary location. We hypothesized that this situation would be different in developing cells wherein extensive chromatin remodeling depends on the availability of TCA cycle metabolites (*5*, *6*, *48*). We therefore examined the presence of IDH3G, ACO2, and OGDH in mouse embryonic stem (ES) cells by immunofluorescence and detected a clear nuclear signal for the latter two enzymes (Fig. 4C and fig. S7). Notably, upon induction of differentiation, a reorganization of the nuclear and mitochondrial pattern of OGDH was observed, with the differentiating cells displaying a reduction in the nuclear OGDH levels (Fig. 4D). To assess the functional relevance of OGDH redistribution, we examined the localization of proteins that are succinylated in their lysine residues. Pan-succinylation in ES cells in the naïve and differentiating state followed the same topological pattern as OGDH, with the differentiating cells exhibiting a reduced nuclear signal with a concomitant increase in the mitochondria (Fig. 4E). Collectively, these results provide a hitherto unrecognized spatial context for the link between metabolism and the epigenetic modulations that underline the differentiation and renewal of ES cells.

## DISCUSSION

Together, our results show that not only specific enzymatic steps but also more extended TCA cycle subnetworks operate in the mammalian nuclei. Proximity of the involved enzymes to canonical nuclear proteins with roles in histones and nucleic acid modifications is in accord with the “on-site” production of critical metabolites. This interpretation is further strengthened by the fact that cells under developmental context exhibited a more prominent nuclear presence of key enzymes than *HeLa* cells. While our data show the metabolic flux activity, it is possible that the nuclear TCA cycle enzymes have additional, so-called moonlighting, functions in the nucleus (*49*). Further investigations using, for example, nucleus localization–defective mutants and quantification of relative activity in different compartments, including measurements of α-ketoglutarate and succinyl-CoA, would be required to disentangle the metabolic and other functions. Nevertheless, our findings challenge the notion confining the TCA cycle to mitochondria and bring forward the distribution of the metabolic activity between mitochondria and nucleus. The here-identified nuclear pathways use citrate and glutamine/glutamate as source molecules and produce downstream metabolites known to be essential for (re)modifications of chromatin and RNA, suggesting a contribution to gene expression regulation.

## MATERIALS AND METHODS

### Nuclei isolation

*HeLa* Kyoto cells (RRID: CVCL_1922) were maintained under standard cultivation conditions (37°C, 5% CO_2_) in Dulbecco’s modified Eagle’s medium (DMEM; high glucose, GlutaMAX, Thermo Fisher Scientific, 61965026) supplemented with 10% heat-inactivated fetal bovine serum (FBS; Thermo Fisher Scientific, 26140079). For nuclei experiments, the cells were seeded in 245 mm by 245 mm plates (2.0 × 10^6^ cells per plate) and cultivated for 3 days. The nuclei isolation method was adapted from Ori and colleagues (*50*). The below described quantities refer to the collection of one 245-mm plate. Briefly, cells were washed three times with 10 ml of phosphate-buffered saline (PBS) followed by addition of trypsin-EDTA (Thermo Fisher Scientific, 25300096). Once cells were detached from the plate, 20 ml of cultivation medium was added, and the cells were collected. From here onward, all steps were performed under ice-cold conditions. Following centrifugation at 500*g* for 5 min, the cell pellet was washed with 10 ml of PBS and centrifuged under the same conditions. Subsequently, the cell pellet was resuspended in 7.5 ml of hypotonic buffer A [50 mM tris-HCl (pH 7.5), aprotinin (1 μg/ml) (Carl-Roth, A162.1), and leupeptin (0.5 μg/ml) (Carl-Roth, CN33.3)] and incubated on ice for 30 min. Once the cells were swollen, rapture was achieved using a Dounce homogenizer with pestle B. Cell lysis efficiency was monitored through light microscopy. Once cells were sufficiently lysed, the hypotonic buffer A was adjusted to buffer B [0.25 M sucrose, 50 mM tris-HCl (pH 7.5), 25 mM KCl, 5 mM MgCl_2_, 2 mM dithiothreitol (DTT), aprotinin (1 μg/ml), and leupeptin (0.5 μg/ml)]. A fraction of whole-cell lysates was immediately used for the ^13^C-labeling experiments. The rest of the lysates were centrifuged at 1000*g* for 8 min, and the pelleted nuclei were resuspended in 10 ml of buffer B. Following centrifugation, the nuclei pellet was resuspended in 3 ml of a buffer that consisted of one part buffer B and two parts buffer C [2.3 M sucrose, 50 mM tris-HCl (pH 7.5), 25 mM KCl, 5 mM MgCl_2_, 2 mM DTT, aprotinin (1 μg/ml), and leupeptin (0.5 μg/ml)]. The resuspended nuclei were transferred to ultracentrifuge tubes (Beckman, #3440057), and 1.5 ml of buffer C was slowly placed at the bottom of the tubes with a needle of at least 18 gauge. The nuclei were centrifuged at 21,000*g* for 30 min in a Beckman ultracentrifuge equipped with a SW55Ti rotor. Subsequently, the interphase and the supernatant were carefully aspirated via vacuum, and the nuclei pellet was resuspended in 1 ml of buffer B and centrifuged at 1000*g* for 8 min. This step was repeated two more times. The nuclei were immediately used for the ^13^C-labeling experiments. Alternatively, samples were saved at −80°C for proteomics and immunoblot analyses.

### Nuclear membrane isolation

The isolation of nuclear membranes was adapted from Ori and colleagues (*50*). The starting material is freshly isolated nuclei resuspended in buffer B (see the “Nuclei isolation” section). Following centrifugation (800*g*, for 5 min, at 4°C), the pellet was slowly resuspended in 200 μl of buffer A-NE [0.1 mM MgCl_2_, 2 mM DTT, aprotinin (1 μg/ml), leupeptin (0.5 μg/ml), deoxyribonuclease I (5 μg/ml), and ribonuclease A (50 μg/ml)] and then in 800 μl of buffer B-NE [10% sucrose, 20 mM tris-HCl (pH 8.5), 0.1 mM MgCl_2_, 2 mM DTT, aprotinin (1 μg/ml), and leupeptin (0.5 μg/ml)]. The sample was incubated for 30 min at room temperature while rotating. Buffer C-NE (800 μl) [30% sucrose, 20 mM tris-HCl (pH 7.5), 0.1 mM MgCl_2_, 2 mM DTT, aprotinin (1 μg/ml), and leupeptin (0.5 μg/ml)] was slowly placed at the bottom of the tube with a needle of at least 18 gauge. Following centrifugation (1500*g*, for 15 min, at 4°C), the pellet was washed two times with 1 ml of buffer B. The final pellet that consisted of nuclear membranes was used immediately for the ^13^C-labeling experiments. Alternatively, samples were saved at −80°C for proteomic analysis.

### ^13^C stable isotope tracing experiments

Freshly isolated nuclei were resuspended in 500 μl of incubation buffer containing 0.25 M sucrose, 50 mM tris-HCl (pH 7.5), 25 mM KCl, 5 mM MgCl_2_, 2 mM DTT, aprotinin (1 μg/ml), leupeptin (0.5 μg/ml), 1 mM adenosine 5′-triphosphate (ATP), 1 mM adenosine 5′-diphosphate (ADP), 1 mM flavin adenine dinucleotide (FAD), 1 mM nicotinamide adenine dinucleotide (NAD^+^), and either one of the following substrates at a final concentration of 10 mM: [U-^13^C]pyruvate, [U-^13^C]citrate, [U-^13^C]glutamine, [U-^13^C]succinate, or [1,2-^13^C_2_]acetyl-CoA (Cambridge Isotopes Inc.). For the experiments analyzed by LC-MS, the incubation buffer additionally contained 0.1 mM thiamine pyrophosphate and 0.1 mM CoA. For the indicated experiments, the following inhibitors with the respective final concentrations were used: 0.5 μM fluorocitrate (Merck, F9634), 20 μM fluoroacetate (Merck, 341460), 1 mM AOA (Merck, C13408), and 20 μM GSK864 (Merck, SML1757). The nuclei were incubated for 1 or 5 hours in the dark at 37°C. Following incubation, the nuclei were centrifuged at 1000*g* for 8 min at 4°C, washed with 1 ml of ice-cold PBS, and centrifuged again. This step was repeated two times. The nuclei pellets were saved at −80°C until metabolite extraction for metabolomic analysis.

For whole-cell lysates, the same procedure described for the isolated nuclei with slight modifications was followed. In brief, fresh whole-cell lysates resuspended in buffer B were adjusted to the incubation buffer by adding the missing compounds and were incubated as above. At the end of the incubation period, the samples were saved at −80°C until metabolite extraction for metabolomic analysis.

### Metabolite extraction

Polar metabolites were extracted from the nuclei pellet with the addition of 300 μl of ice-cold methanol (ULC/MS grade, Biosolve, 136841) supplemented with 10 μl of adonitol (50 μm/ml; Alfa Aesar, L03253.06) as an internal standard and incubation for 15 min at 72°C. The methanol/nuclei suspension was further mixed with 300 μl of ice-cold MilliQ H_2_O and centrifuged at 15,000 rpm at 4°C for 10 min. The supernatants were transferred into amber glass vials (Agilent, 5183-2073), dried with the Genevac EZ-2 Plus evaporator (program, hplc fraction; temperature, 30°C), and stored at −80°C until analysis with GC-MS or LC-MS.

For the extraction of polar metabolites from whole-cell lysates, 300 μl of whole-cell lysates in incubation buffer was mixed with 600 μl of ice-cold methanol supplemented with 10 μl of adonitol (50 μm/ml) and was incubated for 15 min at 72°C. Ice-cold MilliQ H_2_O (600 μl) was added to the methanol/whole-cell lysate mixture, and the rest of the steps were performed as indicated for the nuclei experiments.

### GC-MS metabolomic data acquisition and analysis

Dried polar metabolites were derivatized with 40 μl of methoxyamine hydrochloride (20 mg/ml; Alfa Aesar, L08415.14) solution in pyridine (Alfa Aesar, A12005) for 90 min at 37°C, followed by reaction with 80 μl of *N*-methyl-*N*-(trimethylsilyl)trifluoroacetamide (Alfa Aesar, A13141) for 10 hours at room temperature, as justified in (*51*). GC-MS analysis was performed using a Shimadzu TQ8040 GC (triple quadrupole)–MS system (Shimadzu Corp.) equipped with a 30 m × 0.25 mm × 0.25 μm ZB-50 capillary column (Phenomenex, 7HG-G004-11). One microliter of the sample was injected in split mode (split ratio 1:5 for the isolated nuclei; split ratio 1:30 for the whole-cell lysates) at 250°C using helium as a carrier gas with a flow rate of 1 ml/min. GC oven temperature was held at 100°C for 4 min, followed by an increase to 320°C with a rate of 10°C/min, and a final constant temperature period at 320°C for 11 min. The interface and the ion source were held at 280° and 230°C, respectively. The detector was operated both in scanning mode recording in the range of 50 to 600 mass/charge ratio (*m*/*z*) and in MRM mode for specified metabolites. For peak annotation, the GCMSsolution software (Shimadzu Corp.) was used. The metabolite identification was based on an in-house database with analytical standards used to define the retention time (RT), the mass spectrum, and the quantifying ion fragment for each specified metabolite. The ratio of the different mass isotopologs for each metabolite was determined by integrating the area under the curve of the quantifying ion fragments followed by correction for the presence of natural abundant isotopes with the Isotope Correction Toolbox (ICT) (*52*). All peak integrations were manually checked (related to tables S1 to S3).

### LC-MS metabolomic data acquisition and analysis

LC-MS analysis was performed with an Agilent 1290 Infinity LC system coupled to Agilent 6546 LC/Q-TOF (Agilent Technologies). Chromatographic separation was achieved using an Agilent InfinityLab Poroshell 120 HILIC-Z UHPLC guard column (2.1 × 5 mm, 2.7 μm, 821725-947) attached to an Agilent InfinityLab Poroshell 120 HILIC-Z column (2.1 × 150 mm, 2.7 μm, 683775-924). Mobile phase A consisted of 10 mM ammonium acetate in water supplemented with 5 μΜ InfinityLab deactivator additive and adjusted to pH 9 with ammonium hydroxide. Mobile phase B consisted of 10 mM ammonium acetate in 90% acetonitrile. Dried polar metabolites were reconstituted in 80 μl of high-performance liquid chromatography (HPLC)–grade H_2_O, and 1 μl of the sample was injected. The following gradient was used: 90% mobile phase B from 0 to 2 min, 90 to 60% mobile phase B from 2 to 12 min, 60% mobile phase B from 12 to 15 min, 60 to 90% mobile phase B from 15 to 16 min, and 90% mobile phase B from 16 to 24 min. Column temperature was maintained at 30°C. The flow rate was set to 0.25 ml/min.

The Agilent 6546 LC/Q-TOF was operated with the following source parameters: gas temperature, 200°C; gas flow, 10 liters/min; nebulizer, 40 psig; sheath gas temperature, 300°C; sheath gas flow, 12 liters/min; Vcap, 2500 V; nozzle voltage, 0 V; fragmentor, 90 V; skimmer1, 45 V; and octupole RF peak, 750. The instrument was set to acquire over the *m/z* range of 30 to 1000 in negative mode with the MS acquisition rate of 2 spectra/s. The mode of acquisition was profile. Online mass calibration was performed using a second ionization source and a constant flow (2 ml/min) of reference solution (112.9856 and 1033.9881 *m/z*).

For data analysis, the MassHunter Profinder Software (B.08.00, Agilent Technologies) was used. An in-house database with analytical standards for all mentioned metabolites was used for processing the acquired data in terms of *m/z* and RT matching. The features were extracted using the batch isotopolog extraction algorithm with a mass error window of 5 parts per million (ppm) and RT match of ±0.5 min. All peak integrations were manually checked (related to tables S4 to S6).

### NLS motif prediction

To identify potential NLS motifs inside of the protein sequences of interest (related to Fig. 2I), we used a computational screen. For this purpose, we used the sophisticated definitions of NLS motif classes of the ELM database (*53*) and extended the regular expression search pattern in a way that we allowed further matches by relaxing the pattern restrictions outside of the NLS consensus core motif. For the final pattern search, we used a local version of the pattern search algorithm 3of5 (*54*).

### Generation of constructs with the TCA cycle enzymes fused with the biotin ligase and a FLAG tag

For the selected enzymes, a smaller version of the biotin ligase (BioID2) (*39*) and a FLAG epitope were fused at the C terminus of the enzymes. For IDH2, we additionally created a version carrying the biotin ligase and the FLAG tag at the N terminus of the enzyme. Constructs containing the gene of interest fused to the biotin ligase and a FLAG epitope were generated via Gateway cloning technology (Thermo Fisher Scientific). First, we created two destination vectors containing the BioID2 and FLAG for N-terminal fusion and C-terminal fusion. To achieve this, we used two destination vectors with the older version of BioID (*55*) [pcDNA5-pDEST-BioID-FLAG-N-term and pcDNA5-pDEST-BioID-FLAG-C-term, donated by M.B. (*56*), European Molecular Biology Laboratory (EMBL), Heidelberg] and replaced it with the new and smaller version of BioID2 via Gibson assembly (New England Biolabs). Briefly, BioID2 in pcDNA3.1-BioID2-HA plasmid (Addgene, #74224) was polymerase chain reaction (PCR) amplified with primers containing the FLAG epitope for N- or C-terminal integration (namely, FLAG-BioID2 or BioID2-FLAG). pcDNA5-pDEST-BioID-FLAG-N-term and pcDNA5-pDEST-BioID-FLAG-C-term plasmids were amplified by PCR, excluding the BioID-FLAG region and creating appropriate overlapping to the BioID2-FLAG or FLAG-BioID2 end fragments. One microliter of the PCR products (15 ng of BioID2-FLAG or 15 ng of FLAG-BioID2; 7.5 ng of pcDNA5-pDEST---N-term or 10 ng of pcDNA5-pDEST---C-term) was incubated with 2 μl of Gibson assembly reaction mix at 50°C for 60 min. Two microliters of the reaction was diluted with 8 μl of water, and 4 μl of this dilution was used to transform One Shot ccd B Survival 2 T1R competent cells (Thermo Fisher Scientific, A10460). Transformants were selected with chloramphenicol (33 μg/ml; Merck, 220551) and ampicillin (100 μg/ml; Merck, 171255). The entry clones with the genes of interests (PDHB, ACO2, IDH2, IDH3G, IDH1, and OGDH) were purchased from the Human ORFeome Collection (Dharmacon). The final constructs were created by performing the LR recombination reaction between the destination vectors and the entry clones according to the manufacturer’s instructions. In brief, 1 μl of entry clone (~100 ng) was combined with 1 μl of destination vector (~150 ng) and 2 μl of LR Clonase II reaction buffer into a final volume of 10 μl with TE buffer. The reaction was incubated at 25°C for 90 min followed by the addition of 1 μl of proteinase K (Merck, 3115887001) and incubation at 37°C for 10 min. One microliter of the LR reaction was used to transform DH5α competent *Escherichia coli* (Thermo Fisher Scientific, EC0112), and transformants were selected with the appropriate antibiotics. All constructs were extracted using the QIAprep Spin Miniprep Kit (Qiagen). The final constructs were sequence verified by Sanger sequencing at Eurofins Genomics. All vectors, selection antibiotics, and primers are listed in tables S10 to S12.

### Generation of stable inducible Flp-In T-REx HeLa cell lines

Flp-In T-REx *HeLa* cells (*57*) were maintained under standard cultivation conditions (37°C, 5% CO_2_) in DMEM (high glucose, GlutaMAX, Thermo Fisher Scientific, 61965026) supplemented with 10% heat-inactivated FBS (Thermo Fisher Scientific, 26140079) and the addition of zeocin (50 μg/ml; Thermo Fisher Scientific, R25005) and blasticidin (5 μg/ml; Thermo Fisher Scientific, R21001). The constructs containing the TCA cycle enzymes fused to the biotin ligase and a FLAG tag were stably integrated in the Flp-In T-REx *HeLa* cells using the X-tremeGENE 9 DNA Transfection Reagent (Merck) following the manufacturer’s instructions. Briefly, Flp-In T-REx *HeLa* cells were seeded in six-well plates (1.8 × 10^4^ cells per well) in cultivation medium without antibiotics. After 24 hours, the transfection mixture was prepared [consisted of 100 μl of Opti-MEM (Thermo Fisher Scientific, 31985062), 3 μl of X-tremeGENE 9 DNA Transfection Reagent, 100 ng of plasmid with the construct of interest, and 900 ng of pOG44 (Thermo Fisher Scientific, V600520)], incubated for 15 min at room temperature, and added in a dropwise manner to the cells under gentle shaking. On the third day, the cells were expanded via trypsinization (trypsin-EDTA, Thermo Fisher Scientific, 25300096) into 150-mm plates, and the following day, the medium was changed into cultivation medium with the addition of the selection antibiotics blasticidin (5 μg/ml) and hygromycin (200 μg/ml; Thermo Fisher Scientific, 10687010). The following weeks, fresh cultivation medium with the selection antibiotics was added thrice per week. When large colonies were formed, the cells were trypsinized into the same plates, and when 80% confluency was reached, they were prepared for cryopreservation.

The parental and stable Flp-In T-REx *HeLa* cell lines were routinely monitored for mycoplasma contamination. The parental Flp-In T-REx *HeLa* cell line has not been authenticated.

### BioID labeling and cell harvesting

The parental and stable Flp-In T-REx *HeLa* cell lines were seeded in 245-mm plates (2.0 × 10^6^ cells per plate) in cultivation medium (described in the previous section) without antibiotics. The cells were allowed to attach for 24 hours, and then tetracycline (0.13 μg/ml; Merck, T7660) was added in the medium of the stable cell lines for the induction of protein expression. Following 24 hours, both parental and stable cells were supplemented with biotin (13 μM; Merck, B4639) for protein biotinylation. Last, after 24 additional hours, the cells were washed with 20 ml of PBS, detached with 10 ml of trypsin-EDTA, and collected with 20 ml of cultivation medium. The collected cells were centrifuged at 500*g* for 5 min at 4°C, resuspended in 15 ml of PBS (4°C), and centrifuged. Once the supernatant was aspirated, the cell pellets were frozen on dry ice and stored at −80°C until further processing. For each case, four biological replicates were collected.

### Affinity purification of biotinylated proteins

The affinity purification of the biotinylated proteins was adapted from the method developed by Mackmull *et al.* (*56*) with slight modifications. The frozen cell pellets were resuspended in 8 ml of ice-cold lysis buffer with the following composition: 50 mM tris (pH 7.5), 150 mM NaCl, 1% Triton X-100, 0.1% SDS, 1 mM EDTA, 1 mM EGTA, 1 mM phenylmethylsulfonyl fluoride (Merck, P7626), aprotinin (1 mg/ml; Carl-Roth, A162.1), leupeptin (0.5 mg/ml; Carl-Roth, CN33.3), and 250 U of HS-Nuclease (MoBiTec, GE-NUC10700-01). Following resuspension, the samples were incubated for 1 hour at 4°C under constant mild rotation (30 rpm). Subsequently, the cell lysates were sonicated at 4°C for 30 s five times, with 30 s of rest in between, and were further centrifuged at 17,000*g* for 30 min at 4°C to remove any insoluble material. Streptavidin Sepharose High Performance beads (80 μl; GE Healthcare, 17-5113-01) were equilibrated in 1 ml of lysis buffer for 30 min at 4°C under constant mild rotation (30 rpm). The equilibrated beads were centrifuged at 2000*g* for 5 min at 4°C, then transferred to the lysed supernatants, and incubated for 3 hours at 4°C under constant mild rotation (30 rpm). After the incubation, the beads were centrifuged at 2000*g* for 5 min at 4°C, and, lastly, 7.5 ml of the supernatant was discarded. The remaining beads-lysates were transferred to a Spin Column (Pierce, Thermo Fisher Scientific, 69705), washed once with 800 μl of lysis buffer, and then five times with 700 μl of 50 mM ammonium bicarbonate (pH 8.3). After the washing, the column was plugged, and the beads were transferred to a fresh Eppendorf tube with 300 μl of 50 mM ammonium bicarbonate (pH 8.3). The same procedure was repeated two more times to ensure that all beads were collected. One microgram of Sequencing Modified Trypsin (Promega, V5117) was added, and the samples were incubated at 37°C for 16 hours under constant shaking (500 rpm). The following day, 0.5 μg of trypsin was added, and the beads were incubated for two additional hours under the same conditions. Following incubation, the beads were transferred to a Spin Column, and the digested peptides were eluted with 150 μl of 50 mM ammonium bicarbonate (pH 8). This step was performed one additional time. The eluted peptides were dried in a speed-vac and stored at −80°C until further processing. All steps were performed using low-retention tips (TipOne RPT Tips, Starlab) and low–protein binding collection tubes (Thermo Fisher Scientific, 90411).

### Sample preparation for MS of biotinylated peptides

Dried samples were dissolved in 1% formic acid with 4% acetonitrile and subjected to OASIS HLB μElution Plate (Waters) for desalting according to the manufacturer’s instructions. Desalted peptides were reconstituted in 50 mM Hepes (pH 8.5) and labeled with TMT10plex158 Isobaric Label Reagent (Thermo Fisher Scientific) according to the manufacturer’s instructions. For further sample cleanup, an OASIS HLB μElution Plate (Waters) was used. Offline high-pH reverse-phase fractionation was carried out on an Agilent 1200 Infinity HPLC system, equipped with a Gemini C18 column (3 μm, 110 Å, 100 × 1.0 mm, Phenomenex) (*58*).

### MS data acquisition of biotinylated peptides

An UltiMate 3000 RSLC nano LC system (Dionex) was fitted with a trapping cartridge (μ-Precolumn C18 PepMap 100, 5 μm, 300 μm inside diameter × 5 mm, 100 Å) and an analytical column (nanoEase M/Z HSS T3 column 75 μm × 250 mm C18, 1.8 μm, 100 Å, Waters). Trapping was carried out with a constant flow of solvent A (0.1% formic acid in water) at 30 μl/min onto the trapping column for 6 min. Subsequently, peptides were eluted via the analytical column with a constant flow of 0.3 μl/min with increasing percentage of solvent B (0.1% formic acid in acetonitrile) from 2 to 4% in 4 min, from 4 to 8% in 2 min, then 8 to 28% for a further 96 min, and lastly from 28 to 40% in another 10 min. The outlet of the analytical column was coupled directly to a QExactive plus (Thermo Fisher Scientific) mass spectrometer using the Proxeon nanoflow source in positive ion mode.

The peptides were introduced into the QExactive plus via a Pico-Tip Emitter with 360 μm outer diameter × 20 μm inside diameter, 10-μm tip (New Objective), and an applied spray voltage of 2.3 kV. The capillary temperature was set at 320°C. Full mass scan was acquired with mass range of 350 to 1400 *m*/*z* in profile mode in the Fourier transform (FT) with a resolution of 70,000. The filling time was set at a maximum of 100 ms with a limitation of 3 × 10^6^ ions. Data-dependent acquisition was performed with the resolution of the Orbitrap set to 35,000, with a fill time of 120 ms and a limitation of 2 × 10^5^ ions. A normalized collision energy of 32 was applied. A loop count of 10 with count 1 was used, and a minimum automatic gain control (AFC) trigger of 2 × 10^2^ was set. Dynamic exclusion time of 30 s was used. The peptide match algorithm was set to “preferred” and charge exclusion “unassigned”; charge states 1 and 5 to 8 were excluded. MS2 data were acquired in profile mode (*59*).

### MS data analysis of biotinylated peptides

IsobarQuant (*60*) and Mascot (v2.2.07) were used to process the acquired data, which were searched against a UniProt *Homo sapiens* proteome database (UP000005640) containing common contaminants and reversed sequences. The following modifications were included into the search parameters: carbamidomethyl (C) and TMT10 (K) (fixed modification), Acetyl (N-term), Oxidation (M), and TMT10 (N-term) (variable modifications). For the full scan (MS1), a mass error tolerance of 10 ppm and, for tandem MS (MS/MS) (MS2), spectra of 0.02 Da were set. Further parameters were set as follows: trypsin as protease with an allowance of maximum two missed cleavages, a minimum peptide length of seven amino acids, and at least two unique peptides were required for protein identification. The false discovery rate (FDR) on peptide and protein level was set to 0.01.

### Identification of putative interactors for each bait enzyme of TCA cycle

Raw data of IsobarQuant were loaded into R (ISBN 3-900051-07-0). As a quality criterion, only proteins that were quantified with at least two different unique peptides were used for downstream analysis. The “signal_sum” columns of the “proteins”-output sheet from IsobarQuant were cleaned for potential batch effects with limma (*61*) and subsequently normalized with vsn (variance stabilization) (*62*). Missing values were imputed with the impute function (method = “knn”) from the MSnBase package (*63*). To create the overview of the biotinylated proteins significantly associated with each bait enzyme, hereafter termed as overview of the putative interactors (related to Fig. 3A and table S7), the following two comparisons were made. First, each cell line expressing a bait enzyme with the biotin ligase fused at the C terminus was compared to parental cells using limma. A biotinylated protein was considered a putative interactor with FDR ≤ 20% and a fold change of at least 50%. These results were subsequently refined by removing proteins characterized as contaminants in the CRAPome database (*64*) (version 1.1) with the following parameters: organism, *H. sapiens*; cell/tissue type, *HeLa*; epitope tag, BirA*-FLAG; cutoff frequency, detected in more than 6 experiments (of 16 experiments in total), unless they displayed a fold change (engineered Flp-In T-REx *HeLa* to parental Flp-In T-REx *HeLa*) ≥2.5. Second, each cell line expressing a bait enzyme fused with biotin ligase at the C terminus was compared to the cell line expressing IDH2 carrying the biotin ligase at the N terminus. This fusion renders the mitochondrial import signal at the N terminus of IDH2 inaccessible; hence, this engineered enzyme cannot access the mitochondria but rather resides in the cytoplasm (related to table S7) and serves as a supplemental negative control to further eliminate unspecific biotinylated proteins as a result of the exogenous expression of the biotin ligase. A biotinylated protein was considered a putative interactor using the same analysis and criteria as in the first comparison. Last, the overview included only those defined as putative interactors in both comparisons.

To define for each bait enzyme a subset of putative interactors that show significant enrichment as compared to the mitochondrial-only IDH2 enzyme (related to Fig. 3B and table S9), hereafter termed as top putative interactors, we next compared each cell line expressing a bait enzyme with the biotin ligase fused at the C terminus to (i) the parental cells, (ii) the cells expressing IDH2 with the biotin ligase fused at the N terminus, and (iii) the cells expressing the IDH2 carrying the biotin ligase at the C terminus. In each of the three comparisons, a biotinylated protein is considered a putative interactor using the same analysis and criteria as in the above paragraph. Last, for each bait enzyme, the proteins defined as putative interacting proteins in all three comparisons were retained. For the case of IDH2 with the biotin ligase at the C terminus, the three following comparisons were performed: (i) to the parental cells, (ii) to the cells expressing the IDH2 with the biotin ligase fused at the N terminus, and (iii) to the cells expressing ACO2 with the biotin ligase fused at the C terminus.

### Heatmap and clustering analysis

Clustering analysis (related to Fig. 3A) was performed for the overview of the putative interactors associated with each bait enzyme (table S7). The input values for each putative interactor corresponded to the average (across all biological replicates) log_2_ fold changes in the abundance of the putative interactor in cells expressing a bait enzyme relative to the parental *HeLa* cells. Hierarchical clustering was performed on the centered and scaled input values with the “eclust” package (*65*) using the “ward.D2” linkage and the “Euclidean” distance metric. For visualization, the “ComplexHeatmap” package (*66*) was used in R.

### GO enrichment analysis

The five major clusters of the putative interactors defined by the hierarchical clustering (related to Fig. 3A) were subjected to GO enrichment analysis (related to tables S7 and S8) by using g:Profiler (version 0.1.9) (*67*). The following parameters were defined: organism, “hsapiens”; significance threshold correction method, “g:SCS”; and user threshold, “0.05.” The statistical domain scope was set to “custom,” and the entire set of detected putative interacting proteins was used as a background list. For the case of IDH2 carrying the biotin ligase at the N terminus, GO enrichment analysis was performed using the “annotated” genes for the statistical domain scope since the custom list did not result in statistically significant results.

### Topological distribution of the biotinylated proteins

To define the topological distribution (related to Fig. 3B) of the top putative interactors for each of the bait enzymes (table S9), we retrieved information from the UniProt database (*68*) (downloaded on 14 September 2020) and the Human Protein Atlas (*35*) (version 19.3, available from www.proteinatlas.org). UniProt was queried for (i) the subcellular localization manual assignments and (ii) for the GO annotations for the term “cellular component.” The input data corresponded to the respective reviewed entries for *H. sapiens*. Human Protein Atlas was queried on the subcellular location data. “Nucleus” and “mitochondria” refer to proteins only detected in the nucleus and mitochondria, respectively. “Nucleus shared” refers to proteins that are detected in the nucleus and any other compartment. This category also includes the proteins that are detected in the nucleus and mitochondria. “Mitochondria shared” refers to proteins that are detected in the mitochondria and any other compartment excluding the nucleus. “Other” includes the proteins that are detected in any compartment other than the nucleus or the mitochondria.

### Dot plot generation

For the dot plot generation (related to Fig. 3E), the ProHits-viz platform (*69*) was used for the top putative interactors (table S9). The input data corresponded to (i) the average (across all biological replicates) log_2_ fold changes in the abundance of each biotinylated protein in the cells expressing a bait enzyme relative to the parental *HeLa* cells, and (ii) the respective *P* values estimated with limma analysis. The following parameters were used: score type, *P* value defined by limma analysis; primary filter, 0.01; and secondary filter, 0.1; no clustering was used.

### Sample preparation for MS proteomic analysis of whole-cell lysates, isolated nuclei, and nuclear membranes

Whole-cell lysates, isolated nuclei, and nuclear membranes were isolated as described earlier. For whole-cell lysates and isolated nuclei, proteins were precipitated with a solution of TCA (at a final concentration of 10%) and β-mercaptoethanol (at a final concentration of 0.07%) by incubating at 4°C for at least 1.5 hours or overnight, followed by centrifugation at 10,000 rpm for 20 min. The pellets were washed thrice with a solution of acetone and β-mercaptoethanol (at a final concentration of 0.07%), and the samples were dried. For total protein amount estimation, the samples were resuspended in 100 μl of 25 mM ammonium bicarbonate followed by the addition of 2 μl of RapiGest 1%, incubation at 80°C for 10 min under agitation (700 rpm), and measure of the protein content with the Pierce 660-nm assay (catalog number 22662). For in-solution digestion, the corresponding volume to 50 μg of total protein was reduced with DTT (final concentration of 4 mM) at 60°C for 10 min, followed by alkylation with iodoacetamide (at a final concentration of 14 mM) at room temperature for 30 min, and quenching with DTT (final concentration of 7 mM). Two hundred nanograms of trypsin was added, and the samples were incubated at 37°C for 16 hours under constant shaking (500 rpm).

### MS proteomic data acquisition of whole-cell lysates, isolated nuclei, and nuclear membranes

Injected samples were analyzed using an Ultimate 3000 RSLC nano system (Thermo Fisher Scientific, Hemel Hempstead) coupled to an Orbitrap Eclipse mass spectrometer (Thermo Fisher Scientific) equipped with FAIMS Pro for gas phase separation of peptides. The sample was loaded onto the trapping column (Thermo Fisher Scientific, PepMap100, C18, 300 μm × 5 mm), using partial loop injection, for 3 min at a flow rate of 15 μl/min with 0.1% (v/v) FA in 3% acetonitrile. The sample was resolved on the analytical column (Easy-Spray C18 75 μm × 500 mm, 2-μm column) at a flow rate of 300 nl/min using a gradient of 97% A (0.1% formic acid), 3% B (80% acetonitrile and 0.1% formic acid) to 25% B over 52 min, then to 40% B for an additional 8 min, and then to 100% B for another 10 min, which remained at 100% B for 13 min, and the percentage of B was then lowered to 3.8% to allow the column to reequilibrate for 15 min before the next injection. Data were acquired using two FAIMS cv’s (−50v and −70v), and each FAIMS experiment had a maximum cycle time of 1.5 s. For both FAIMS experiments, the data-dependent program used for data acquisition consisted of a 120,000-resolution full-scan MS scan [AGC set to 100% (4 × 10^5^ ions) with a maximum fill time of 50 ms]. MS/MS was performed at a resolution of 15,000 [AGC set to 100% (5 × 10^4^ ions) with a maximum fill time of 22 ms] with an isolation window of 1.2 *m*/*z* and a higher-energy collisional-induced dissociation (HCD) collision energy of 30%. To avoid repeated selection of peptides for MS/MS, the program used a 40-s dynamic exclusion window.

### MS proteomic data analysis of whole-cell lysates, isolated nuclei, and nuclear membranes

Raw data were imported and data processed in Proteome Discoverer v2.5 (Thermo Fisher Scientific). The raw files were submitted to an iterative database search using Proteome Discoverer with SequestHF and Inferys rescoring algorithm against the *H. sapiens* database containing human protein sequences from UniProt/Swiss-Prot. Common contaminant proteins [several types of human keratins, bovine serum album (BSA), and porcine trypsin] were added to the database. The spectra identification was performed with the following parameters: MS accuracy, 10 ppm; MS/MS accuracy of 0.02 Da for spectra acquired in Orbitrap analyzer; up to two missed cleavage sites allowed; carbamidomethylation of cysteine as a fixed modification; and oxidation of methionine as variable modifications. Percolator node was used for FDR estimation, and only rank 1 peptide identifications of high confidence (FDR < 1%) were accepted. For comparison, whole-cell lysates, isolated nuclei, and isolated nuclear membranes were normalized to LMNB1.

### Immunofluorescence labeling

For the immunofluorescence analysis of the engineered Flp-In T-REx *HeLa* expressing a bait enzyme (related to Figs. 3, C and D, and 4A and figs. S3 to S5), cells were seeded in coverglass chambers (Nunc Lab-Tek II, Merck, Z734853) and allowed to attach for 24 hours before tetracycline addition (0.13 μg/ml; Merck, T7660) for the induction of protein expression. After 24 hours, biotin (13 μM; Merck, B4639) was added for protein biotinylation. Last, after 24 additional hours, the cells were washed with PBS and fixed for 10 min with 4% formaldehyde (Image-iT fixative solution, Thermo Fisher Scientific, FB002) at room temperature. Following three washing steps with PBS, the cells were permeabilized for 20 min with 0.2% Triton X-100 in PBS, washed thrice with PBS, and blocked (0.1% BSA, 0.3 M glycine, and 0.1% Tween 20, in PBS) for 1 hour at room temperature. Incubation with primary antibodies (0.3 M glycine and 0.1% Tween 20, in PBS) was performed overnight in a humidified dark chamber at 4°C. The following antibodies and dilutions were used: streptavidin conjugated to Alexa Fluor 488 (1:100; Thermo Fisher Scientific, S11223), rabbit anti-OGDH (1:100; Merck, HPA020347), rabbit anti-IDH3G (1:100; Merck, HPA002017), rabbit anti-ACO2 (1:100; Abcam, ab129069), rabbit anti-IDH2 (1:100; Merck, HPA007831), and mouse anti-Tom20 (1:100; BD Biosciences, 612278). The next day, the cells were washed three times with PBS and 0.1% (v/v) Tween 20 (PBST) and incubated with secondary antibodies (0.3 M glycine and 0.1% Tween 20, in PBS) for 1 hour in a humidified dark chamber at room temperature. The following secondary antibodies and dilutions were used: anti-rabbit Alexa Fluor 647 (1:200; Thermo Fisher Scientific, A27040) and anti-mouse Alexa Fluor 555 (1:200; Thermo Fisher Scientific, A28180). Following three washing steps with PBST, the cells were stained with Hoechst (2.6 μg/ml; Thermo Fisher Scientific, 62249) for 20 min in the dark at room temperature. Last, the cells were washed three times with PBS and saved in PBS supplemented with 0.02% sodium azide (Merck, S2002) at 4°C until image acquisition.

For the immunofluorescence analysis of cell lysates and isolated nuclei (related to Fig. 4B and fig. S6), engineered Flp-In T-REx *HeLa* cells were grown and treated with tetracycline and biotin as before, followed by the nuclei isolation procedure described above. Cell lysates and isolated nuclei were resuspended in buffer [0.25 M sucrose, 50 mM tris-HCl (pH 7.5), 25 mM KCl, 5 mM MgCl_2_, 2 mM DTT, leupeptin (10 mg/ml), aprotinin (5 mg/ml)] and were attached via centrifugation on coverslips (thickness #1.5, Thermo Fisher Scientific, CB00110RAC20MNT0) previously coated with poly-l-lysine (Thermo Fisher Scientific, P4832). The rest of the steps were the same as described for the immunofluorescence analysis of whole cells with the following modifications: Primary and secondary antibody incubation buffer consisted of 0.3 M glycine and 0.025% Tween 20 in PBS, Hoechst staining was performed for 10 min, and all washes were done with PBS. Last, all coverslips were mounted on slides with one drop of ProLong Gold Antifade mountant (Thermo Fisher Scientific, P10144), dried in the dark overnight at room temperature, and saved at −20°C until image acquisition. The following primary and secondary antibodies along with the respective dilutions were used: streptavidin conjugated to Alexa Fluor 488 (1:100; Thermo Fisher Scientific, S11223), mouse anti-lamin B1 (1:500; Atlas Antibodies, AMAb91251), and anti-mouse Alexa Fluor 555 (1:1000; Thermo Fisher Scientific, A28180).

For the immunofluorescence analysis of mouse ES cells (related to Fig. 4, C to E, and fig. S7), cells were grown on mouse embryonic fibroblasts (MEFs) in KnockOut DMEM medium (Gibco, 10829018) supplemented with 1% penicillin-streptomycin (Gibco, 15070063), 1% l-glutamine (Gibco, 25030081), 1% MEM Non-Essential Amino Acids Solution (Gibco, 11140050), 15% FBS (Gibco, 10270106), leukemia inhibitory factor (LIF; 0.024 μg/ml; EMBL Protein Expression and Purification Core Facility), and 0.12 mM β-mercaptoethanol (Gibco). Cells were maintained on MEFs and incubated at 37°C with 5% CO_2_ and 95% relative humidity. TrypLE Express Enzyme (1×) (Gibco, 12605036) was used to detach cells for passaging, collection, or to create a single-cell suspension. For immunofluorescence staining, 31,600 ES cells per well were grown without feeders on chambers (ibidi, μ-slide 8 well, #80826) previously coated with 0.1% gelatin in 300 μl of growth medium. To differentiate mouse ES cells (*70*), the cells were passaged twice on 0.1% gelatin to remove MEFs first in DMEM-ES medium and then using IMDM-ES medium containing IMDM (Iscove’s modified Dulbecco medium) (Lonza; BE12-726F) supplemented with 1% penicillin-streptomycin and 1% l-glutamine. In addition, 15% FBS (Gibco, 10270106), LIF (0.024 μg/ml), and 0.12 mM β-mercaptoethanol were added. Following these gelatin passages, ESCs were cultured in untreated 10-cm^2^ petri dishes at a density of 0.3 × 10^6^ cells per dish with EB medium containing IMDM (supplemented with 1% penicillin-streptomycin and 1% l-glutamine), 10% FBS (Gibco, 10270106), 0.6% transferrin (Roche, 10652), 0.03% monothioglycerol (MTG; Sigma-Aldrich, M6145), and ascorbic acid (50 μg/ml; Sigma-Aldrich, A4544). After 3 days in culture, differentiated ESCs expressing Flk1 (hematopoietic mesodermal cells) were isolated through magnetic-activated cell sorting (*70*) using an anti-Flk1 allophycocyanin (APC)–conjugated antibody (eBiosciences, 17-5821-81) and anti-APC microbeads (Miltenyi Biotec, 130-090-855). Flk1^+^ cells were further differentiated at a density of 26,316 cells per well for immunofluorescence analysis on gelatin hours in IMDM medium supplemented with 1% penicillin-streptomycin, 1% l-glutamine, 15% FBS (Gibco, 10270106), transferrin (Roche), MTG (Sigma-Aldrich), ascorbic acid (50 mg/μl; Sigma-Aldrich), vascular endothelial growth factor (10 μg/ml; Preprotech, 500-P131), and interleukin-6 (10 μg/ml; Preprotech, 216-16). After 48 hours, ES cells and differentiated ESCs were subjected to immunofluorescence staining following the same procedure as for the engineered Flp-In T-REx *HeLa* cells. For the detection of succinyl-lysine residues, the rabbit anti-succinyllysine (1:100; PTM-401, PTM Biolabs) and anti-rabbit Alexa Fluor 546 (1:200; Thermo Fisher Scientific, A-11010) were used.

### Image acquisition and analysis

Images of the engineered Flp-In T-REx *HeLa* cells (related to Figs. 3, C and D, and 4A and figs. S3 to S5) were acquired as single optical sections using the same settings for all conditions at the Advanced Light Microscopy Facility at EMBL, Heidelberg, with a Leica TCS SP8 system and a 63×/1.40 oil objective (HC PL APO CS2). To capture the fluorophores and eliminate any cross-talk, the following three sequential acquisition settings with the respective wavelength excitation (ex) and emission (em) detection windows were used: Nr.1, for Hoechst and Alexa Fluor 647, ex:405/em:420–490 nm and ex:638/em:650–750 nm, respectively; Nr.2, for Alexa Fluor 488, ex:488/em:500–531 nm; and Nr.3, for Alexa Fluor 555, ex:552/em:562–592 nm. Scan speed was set at 100 Hz, line averaging at 5, and image resolution was 3144 × 3144 pixels. The image files were subsequently processed with ImageJ/Fiji (National Institutes of Health) software (*71*). Briefly, nuclei were located based on the Hoechst staining, and a region of interest (ROI) inside each nucleus was manually selected to ensure that only pixels inside the nucleoplasm are measured. Next, a ROI covering the nucleus and an extended area around was manually created delineating the “whole cell.” Last, staining based on Tom20, an outer mitochondrial membrane protein, was used to segment the mitochondria ROIs. The biotinylation levels were quantified in whole cells (related to Fig. 3D and fig. S3B), mitochondria regions (related to fig. S3C), and the nuclei regions (related to Fig. 4A) based on the mean intensity values of Alexa Fluor 488–conjugated streptavidin staining. To quantify the enzyme levels, the mean intensity of the enzyme was measured in the “whole-cell” regions (related to Fig. 3C and fig. S3A). The mean intensity values were examined for statistical significance with the Wilcoxon rank sum test. For visualization purposes, all images were treated using the same intensity range (minimum/maximum).

Images of lysed cells and isolated nuclei from the engineered Flp-In T-REx *HeLa* cells (related to Fig. 4B and fig. S6) were acquired as described above with the modification that Nr.1 acquisition setting included only the Hoechst wavelength excitation and emission detection window, and image analysis was performed only for the nucleus ROIs. To account for possible technical variations, we further normalized the biotinylation levels in each individual ROI to the corresponding mean intensity values of the Hoechst staining. Statistical analysis and visualization were performed as described in the section above.

Images of mouse ES cells (related to Fig. 4C and fig. S7) were acquired in the form of z-stacks (step size of 0.34 μm) using the same settings for all conditions with a confocal Leica TCS SP5 microscope and a 40.0× 1.25 oil objective (HC PL Apo UV optimized). The following two sequential acquisition settings with the respective wavelength excitation (ex) and emission (em) detection windows were used: Nr.1, for Hoechst, ex:405/em:420–612 nm; Nr.2, for Alexa Fluor 647, ex:638/em:644–800 nm. Scan speed was set at 100 Hz, and image resolution was 1024 × 1024 pixels. The three-dimensional (3D) image reconstruction and downstream analysis were performed with the Imaris v.9.5.1 (Bitplane) software. In brief, the “surfaces” function was used with the Hoechst staining as the source channel to create 3D surfaces demarcating the nuclei. To ensure that the regions covered mainly the nucleoplasm, a high threshold value (absolute threshold option set at 100; smoothing option disabled) was selected. Subsequently, the mean intensity values of the fluorescent channel corresponding to the enzyme staining (Alexa Fluor 647) were measured within the 3D nuclei surfaces (related to Fig. 4C and fig. S7, A and C). To quantify the enzyme levels in the mitochondria (related to fig. S7B), we used the “spots” function for the enzyme staining channel. The different spot sizes option was selected, the diameter was set at 1 μm, and background subtraction was enabled. To capture the spots relevant to the mitochondria regions, we used the mean intensity of the source channel as the filter type with a high threshold value (*50*). Last, the sensitivity for defining the spots was further adjusted using the “spots region from local contrast” option and a high threshold value (100). The mean intensity values were examined for statistical significance with the Wilcoxon rank sum test. For visualization purposes, one representative plane is depicted, while all images were treated using the same intensity range (minimum/maximum).

Images of mouse ES cells, in naïve state or following differentiation, for OGDH and pan-succinyllysine (related to Fig. 4, D and E, and fig. S7, D and E) were acquired in the form of z-stacks using the same settings for all conditions with a Carl Zeiss LSM880 Axio Observer microscope and a 63× 1.46 oil objective (alpha Plan-Apochromat). The following three sequential acquisition settings with the respective wavelength excitation (ex) and emission (em) detection windows were used: Nr.1, for Alexa Fluor 488, ex:488/em:508–535; Nr.2, for Hoechst, ex:405/em:410–490; and Nr.3, for Alexa Fluor 546, ex:561/em:566–679. Image resolution was 1024 × 1024 pixels. Image analysis was performed as described in the previous paragraph. For visualization purposes, one representative plane is depicted, while all images were treated using the same intensity range (minimum/maximum).

### shRNA-mediated knockdown of ACO2

shRNA constructs targeting human ACO2 were purchased from Origene (TL314993), and *HeLa* cells were transfected using the calcium phosphate transfection method. Briefly, *HeLa* cells were seeded at 30% confluency 2 hours before transfection. For one 245-mm dish, 200 μg of plasmid was mixed with CaCl_2_ (125 mM final concentration) and subsequently dropwise added to Hepes-buffered saline (HBS). The plasmid-CaCl_2_-HBS mix was incubated for 30 min at room temperature and then added to the cells. Following 24 hours, the procedure was repeated. The cells were lastly harvested after 24 hours for the isolation of nuclei and the performance of [U-^13^C]citrate labeling experiments (related to Fig. 2G).

### Localization of succinylated peptides by isotope tagging

Experiments to determine the subcellular localization of unmodified and succinylated peptides (related to Fig. 3F) were performed using the LOPIT-DC protocol (*47*), with the following modifications. Cell lysis buffer consisted of 0.25 M sucrose, 10 mM Hepes (pH 7.4), 2 mM EDTA, 2 mM magnesium acetate, cOmplete mini EDTA-free Protease Inhibitor (Roche), Phosphatase Inhibitor (PhosStop Roche), and Deacetylation Inhibition Cocktail (Santa Cruz) supplemented with 5 μM suramin sodium (Sigma-Aldrich). Cell lysis was performed with a ball-bearing homogenizer (Isobiotec) where each 1.5 ml of cells was passed through a homogenizer chamber 25 times with 12 μm of ball-bearing clearance size. No “clearance” spin at 200*g* was performed. Instead, cell lysates were immediately subjected to the following centrifugation speeds at 4°C, with supernatants removed and subjected to further centrifugations to yield nine pellet fractions and one final supernatant fraction: 100*g* (10 min), 500*g* (5 min), 1000*g* (10 min), 3000*g* (10 min), 5000*g* (10 min), 9000*g* (15 min), 15,000*g* (15 min), 30,000*g* (20 min), and 120,000*g* (45 min). Collected fractions were stored at −20°C. Pellets were solubilized using a membrane solubilization buffer that contained 8 M urea, 0.2% (w/v) SDS, and 50 mM Hepes (pH 8.5). Protein concentration quantitation was performed with a BCA kit (Thermo Fisher Scientific) according to the manufacturer’s instructions and precipitated with ethanol/acetone overnight to remove urea and protease inhibitors that could interfere with protein digestion by the addition of ice-cold ethanol and ice-cold acetone at 4:4:1 ratio (v:v:v) to the fraction, with vortex and incubation at −20°C overnight. Samples were then centrifuged at 16,000*g* for 15 min (4°C), and the supernatant was discarded. Precipitated subcellular fractions were solubilized in 100 to 400 μl of 100 mM TEAB and reduced with 15 mM DTT for 1 hour at 37°C followed by alkylation with 55 mM iodoacetamide for 1 hour at room temperature in the dark. Two-step digestion was performed, first with 3 μg of trypsin (Promega) overnight at 37°C followed by further digestion with 1 μg of trypsin for 4 hours. Fractions were acidified with trifluoroacetic acid (0.1% final concentration), centrifuged to pellet any debris, and peptide supernatant was desalted. For peptide cleanup, peptide desalting spin columns (Pierce) were used, according to the manufacturer’s guideline. Collected peptides from the 10 subcellular fractions were quantified with Qubit (manufacturer’s details), and 80 μg of peptides from each fraction was taken and dried before TMT labeling according to the manufacturer’s guidelines. TMT-labeled fractions were combined in equal proportions (multiplexed). Ten percent of each multiplex was removed for the total proteome analysis. Succinylated peptides were enriched from the remaining 90%. Peptides were first desalted with Peptide Desalting Spin Column (Pierce) according to the manufacturer’s guideline and dried before enrichment using a PTMScan Succinyl-Lysine Motif kit (Cell Signaling Technologies; batch number 13605BC) following the manufacturer’s guidelines, except that the sample and antibody-bead were incubated for 24 hours instead of 2 hours. Alkaline reverse-phase prefractionation of the total proteome was performed as detailed in (*47*). For downstream MS analysis, the fractions corresponding to each TMT 10-plex set were orthogonally combined into 15 samples by combining pairs of fractions that eluted at different time points during the gradient. These fractions were dried down and solubilized in 0.1% formic acid where approximately 1.5 μg of peptides was loaded per MS run.

### LOPIT mass spectrometry

For the total proteome fractions, peptide quantification by SPS-MS3 on the Orbitrap Fusion Lumos was performed as described in (*47*), except that the orbitrap resolution for SPS ions was set to 50,000, and the AGC target was set to 200,000. For the succinyl-enriched sample, data were acquired in a positive ion mode, with a 240-min run, using the following chromatography gradient: 3 min at 2% solvent B, 212-min linear gradient of 2 to 40% solvent B, 0.3-min linear gradient of 40 to 90% solvent B, 9.7 min at 9 0% solvent B, 0.3-min linear gradient of 90 to 2% buffer B, and 14.7 min at 2% buffer B, where solvent A was 0.1% (v/v) formic acid and solvent B was 80% (v/v) acetonitrile + 0.1% (v/v) formic acid. Ions were measured as per the total proteome fractions except that the MS1 scan range was *m*/*z* 400 to 1400 Da, and precursor ions were fragmented using HCD (normalized collision energy of 32%). The succinyl-enriched sample was injected twice in the mass spectrometer.

### LOPIT data processing and analysis

Raw data processing was performed as described in (*47*), except using Proteome Discoverer v.2.4 (Thermo Fisher Scientific), and except that MS/MS accuracy was set to 0.5 Da, and for succinyl peptide-enriched sample, succinylation of lysine was included as variable modifications. Raw files were searched against the reference *H. sapiens* database (UP000005640; downloaded from www.uniprot.org, April 2018) and common contaminant from the common Repository of Adventitious Proteins (cRAP) v1.0 (48 sequences, adapted from the Global Proteome Machine repository, www.thegpm.org/crap/), using Proteome Discoverer with SequestHF algorithm. The succinyl-enriched sample technical replicas were combined into a single output.

Data processing and analysis from the peptide spectrum match (PSM)–level output from Proteome Discoverer are presented in the succinyl-LOPIT GitHub repository github.com/CambridgeCentreForProteomics/succinyl_LOPIT (archived with DOI 10.5281/zenodo.5722391). PSM filtering and summarization to peptide-level quantification were performed in R (version 4.0.3) using MSnBase (*72*), pRoloc (*73*), tidyverse (*74*), and camprotR (github.com/CambridgeCentreForProteomics/camprotR) packages. In brief, PSMs were removed if they matched a cRAP protein, did not have an assigned “master” protein, did not have quantification values, and had signal:noise ratio less than 5 or coisolation/interference above 50%. For the succinyl multiplex, peptides without a succinyl site with site probability scores (ptmRS) above 0.75 were also removed. Up to three missing values were imputed using KNN (*k* = 10) with sum normalization beforehand and denormalization after imputation to ensure that nearest neighbors had similar profiles over the tags rather than a similar overall intensity. PSM intensities were then log center-median normalized before exponentiating back to the untransformed values. PSMs from the total multiplex (without succinyl enrichment) were aggregated to peptide-level and protein-level intensities by summation of PSM-level tag intensities. Similarly, PSMs from the succinyl-enriched multiplex were aggregated to succinyl-peptide level by summation of PSM-level tag intensities. Peptide- and protein-level quantifications were row-sum normalized. Markers used in (*47*) were then annotated, with peptide markers being all detected peptides from each marker protein. Support vector machine (SVM)-based prediction of peptide localization was performed as described in (*47*), with the exception that the training was performed on the unmodified marker peptides only, and the subsequent classification was performed on all peptides (unmodified and succinylated). Thirty rounds of fivefold cross-validation were used to determine the optimal parameters, and the top quartile of assignments for each localization were retained.

To detect overrepresented GO terms in the succinylated nuclear proteins, all proteins with a detected nuclear peptide were annotated as succinylated if at least one succiny-peptide from the protein was detected in the nucleus. Overrepresentation of GO terms was then determined using GOseq (*75*) with the overall protein abundance in (*76*) being the bias factor. *P* values were adjusted to account for multiple testing using the Benjamini-Hochberg procedure (*77*), and GO terms with adjusted *P* values <0.01 (estimated FDR < 1%) were retained. Redundant GO terms were excluded using camproR::remove_redundant_go to leave a representative set of significantly enriched GO terms.

### Immunoblotting

For immunoblotting (related to Fig. 1B and figs. S9 to S12), isolated nuclei were collected as described in the respective section. For whole-cell samples, *HeLa* cells were collected with trypsinization, and the cell pellets were stored at −80°C until analysis. The samples were lysed in SDS–polyacrylamide gel electrophoresis sample buffer [62.5 mM tris (pH 6.8), 2% SDS, 10% glycerol, 0.0006% bromophenol blue, and 5% β-mercaptoethanol], incubated at 95°C for 5 min, and, lastly, sonicated to shear the DNA. The proteins were separated on 4 to 20% gradient gels (Mini-PROTEAN TGX Precast Protein Gels, Bio-Rad). Before transfer, the gel, the nitrocellulose membrane (Thermo Fisher Scientific, 88018), and the blot filter papers (Bio-Rad, #1703932) were equilibrated in transfer buffer [25 mM tris, 192 mM glycine (pH 8.3), 20% methanol, and 0.04% SDS] for 15 min with agitation. For the transfer, the Trans-Blot Turbo System (Bio-Rad) with the “STANDARD SD” protocol followed by the “Mixed MW” program was used. The membrane was stained with Amido Black solution (Merck, A8181) for 10 min and imaged in a ChemiDoc MP Imaging System (Bio-Rad). Following destaining with water, the membrane was blocked with 3% BSA in PBST for 1 hour at room temperature. The primary antibodies were diluted in 5% BSA in PBST, and the membrane was incubated overnight at 4°C under agitation. The following day, the membrane was washed thrice with PBST, incubated for 1 hour at room temperature with the appropriate secondary antibodies in 3% BSA in PBST, and then washed thrice with PBST. Last, the membrane was developed using the Pierce ECL Plus Western Blotting Substrate (Thermo Fisher Scientific, 32132) and imaged in a ChemiDoc MP Imaging System (Bio-Rad). The following antibodies and dilutions were used: rabbit anti-IDH2 (1:1000; Merck, HPA007831), rabbit anti-COXIV (1:1000; Cell Signaling Technology, #8674), rabbit anti–cytochrome c (1:1000; Cell Signaling Technology, #8674), rabbit anti–beta III tubulin (1:1000; Abcam, ab18207), and anti-rabbit horseradish peroxidase linked (1:2000; Abcam, ab205718). As a molecular weight marker, we used the Precision Plus Protein Prestained Standard in Dual Color (Bio-Rad, 1610374).

## Notes

View/request a protocol for this paper from *Bio-protocol*.

## References

[1] L. Cai, B. M. Sutter, B. Li, B. P. Tu, Acetyl-CoA induces cell growth and proliferation by promoting the acetylation of histones at growth genes. Mol. Cell 42, 426–437 (2011). 21596309 10.1016/j.molcel.2011.05.004 PMC3109073

[2] M. Xiao, H. Yang, W. Xu, S. Ma, H. Lin, H. Zhu, L. Liu, Y. Liu, C. Yang, Y. Xu, S. Zhao, D. Ye, Y. Xiong, K. L. Guan, Inhibition of α-KG-dependent histone and DNA demethylases by fumarate and succinate that are accumulated in mutations of FH and SDH tumor suppressors. Genes Dev. 26, 1326–1338 (2012). 22677546 10.1101/gad.191056.112 PMC3387660

[3] A. M. Cervera, J. P. Bayley, P. Devilee, K. J. McCreath, Inhibition of succinate dehydrogenase dysregulates histone modification in mammalian cells. Mol. Cancer 8, 89 (2009). 19849834 10.1186/1476-4598-8-89 PMC2770992

[4] M. Sciacovelli, E. Gonçalves, T. I. Johnson, V. R. Zecchini, A. S. H. Da Costa, E. Gaude, A. V. Drubbel, S. J. Theobald, S. R. Abbo, M. G. B. Tran, V. Rajeeve, S. Cardaci, S. Foster, H. Yun, P. Cutillas, A. Warren, V. Gnanapragasam, E. Gottlieb, K. Franze, B. Huntly, E. R. Maher, P. H. Maxwell, J. Saez-Rodriguez, C. Frezza, Fumarate is an epigenetic modifier that elicits epithelial-to-mesenchymal transition. Nature 537, 544–547 (2016). 27580029 10.1038/nature19353 PMC5136292

[5] B. W. Carey, L. W. Finley, J. R. Cross, C. D. Allis, C. B. Thompson, Intracellular α-ketoglutarate maintains the pluripotency of embryonic stem cells. Nature 518, 413–416 (2015). 25487152 10.1038/nature13981 PMC4336218

[6] T. TeSlaa, A. C. Chaikovsky, I. Lipchina, S. L. Escobar, K. Hochedlinger, J. Huang, T. G. Graeber, D. Braas, M. A. Teitell, α-Ketoglutarate accelerates the initial differentiation of primed human pluripotent stem cells. Cell Metab. 24, 485–493 (2016). 27476976 10.1016/j.cmet.2016.07.002 PMC5023506

[7] R. Chowdhury, K. K. Yeoh, Y. M. Tian, L. Hillringhaus, E. A. Bagg, N. R. Rose, I. K. H. Leung, X. S. Li, E. C. Y. Woon, M. Yang, M. A. McDonough, O. N. King, I. J. Clifton, R. J. Klose, T. D. W. Claridge, P. J. Ratcliffe, C. J. Schofield, A. Kawamura, The oncometabolite 2-hydroxyglutarate inhibits histone lysine demethylases. EMBO Rep. 12, 463–469 (2011). 21460794 10.1038/embor.2011.43 PMC3090014

[8] W. Xu, H. Yang, Y. Liu, Y. Yang, P. Wang, S. H. Kim, S. Ito, C. Yang, P. Wang, M. T. Xiao, L. X. Liu, W. Q. Jiang, J. Liu, J. Y. Zhang, B. Wang, S. Frye, Y. Zhang, Y. H. Xu, Q. Y. Lei, K. L. Guan, S. M. Zhao, Y. Xiong, Oncometabolite 2-hydroxyglutarate is a competitive inhibitor of α-ketoglutarate-dependent dioxygenases. Cancer Cell 19, 17–30 (2011). 21251613 10.1016/j.ccr.2010.12.014 PMC3229304

[9] M. D. Hirschey, Y. Zhao, Metabolic regulation by lysine malonylation, succinylation, and glutarylation. Mol. Cell. Proteomics 14, 2308–2315 (2015). 25717114 10.1074/mcp.R114.046664 PMC4563717

[10] A. M. Gonzalez-Angulo, T. Iwamoto, S. Liu, H. Chen, K.-A. Do, G. N. Hortobagyi, G. B. Mills, F. Meric-Bernstam, W. F. Symmans, L. Pusztai, Gene expression, molecular class changes, and pathway analysis after neoadjuvant systemic therapy for breast cancer. Clin. Cancer Res. 18, 1109–1119 (2012). 22235097 10.1158/1078-0432.CCR-11-2762 PMC3288822

[11] P. S. Liu, H. Wang, X. Li, T. Chao, T. Teav, S. Christen, G. D. I. Conza, W. C. Cheng, C. H. Chou, M. Vavakova, C. Muret, K. Debackere, M. Mazzone, H. Da Huang, S. M. Fendt, J. Ivanisevic, P. C. Ho, Α-ketoglutarate orchestrates macrophage activation through metabolic and epigenetic reprogramming. Nat. Immunol. 18, 985–994 (2017). 28714978 10.1038/ni.3796

[12] S. Raffel, M. Falcone, N. Kneisel, J. Hansson, W. Wang, C. Lutz, L. Bullinger, G. Poschet, Y. Nonnenmacher, A. Barnert, C. Bahr, P. Zeisberger, A. Przybylla, M. Sohn, M. Tönjes, A. Erez, L. Adler, P. Jensen, C. Scholl, S. Fröhling, S. Cocciardi, P. Wuchter, C. Thiede, A. Flörcken, J. Westermann, G. Ehninger, P. Lichter, K. Hiller, R. Hell, C. Herrmann, A. D. Ho, J. Krijgsveld, B. Radlwimmer, A. Trumpp, BCAT1 restricts αkG levels in AML stem cells leading to IDHmut-like DNA hypermethylation. Nature 551, 384–388 (2017). 29144447 10.1038/nature24294

[13] J. P. Morris, J. J. Yashinskie, R. Koche, R. Chandwani, S. Tian, C.-C. Chen, T. Baslan, Z. S. Marinkovic, F. J. Sánchez-Rivera, S. D. Leach, C. Carmona-Fontaine, C. B. Thompson, L. W. S. Finley, S. W. Lowe, α-Ketoglutarate links p53 to cell fate during tumour suppression. Nature 573, 595–599 (2019). 31534224 10.1038/s41586-019-1577-5 PMC6830448

[14] Y. Jiang, X. Qian, J. Shen, Y. Wang, X. Li, R. Liu, Y. Xia, Q. Chen, G. Peng, S.-Y. Lin, Z. Lu, Local generation of fumarate promotes DNA repair through inhibition of histone H3 demethylation. Nat. Cell Biol. 17, 1158–1168 (2015). 26237645 10.1038/ncb3209 PMC4800990

[15] S. Sivanand, S. Rhoades, Q. Jiang, J. V. Lee, J. Benci, J. Zhang, S. Yuan, I. Viney, S. Zhao, A. Carrer, M. J. Bennett, A. J. Minn, A. M. Weljie, R. A. Greenberg, K. E. Wellen, Nuclear acetyl-CoA production by ACLY promotes homologous recombination. Mol. Cell 67, 252–265.e6 (2017). 28689661 10.1016/j.molcel.2017.06.008 PMC5580398

[16] P. L. Sulkowski, S. Oeck, J. Dow, N. G. Economos, L. Mirfakhraie, Y. Liu, K. Noronha, X. Bao, J. Li, B. M. Shuch, M. C. King, R. S. Bindra, P. M. Glazer, Oncometabolites suppress DNA repair by disrupting local chromatin signalling. Nature 582, 586–591 (2020). 32494005 10.1038/s41586-020-2363-0 PMC7319896

[17] Z. Dai, V. Ramesh, J. W. Locasale, The evolving metabolic landscape of chromatin biology and epigenetics. Nat. Rev. Genet. 12, 737–753 (2020). 10.1038/s41576-020-0270-8 PMC8059378 32908249

[18] Y. Shin, C. P. Brangwynne, Liquid phase condensation in cell physiology and disease. Science 357, eaaf4382 (2017). 28935776 10.1126/science.aaf4382

[19] G. Sutendra, A. Kinnaird, P. Dromparis, R. Paulin, T. H. Stenson, A. Haromy, K. Hashimoto, N. Zhang, E. Flaim, E. D. Michelakis, A nuclear pyruvate dehydrogenase complex is important for the generation of acetyl-CoA and histone acetylation. Cell 158, 84–97 (2014). 24995980 10.1016/j.cell.2014.04.046

[20] W. Y. Shi, X. Yang, B. Huang, W. H. Shen, L. Liu, NOK mediates glycolysis and nuclear PDC associated histone acetylation. Front Biosci (Landmark Ed). 22, 1792–1804 (2017). 28410146 10.2741/4572

[21] J. Chen, I. Guccini, D. Di Mitri, D. Brina, A. Revandkar, M. Sarti, E. Pasquini, A. Alajati, S. Pinton, M. Losa, G. Civenni, C. V. Catapano, J. Sgrignani, A. Cavalli, R. D’Antuono, J. M. Asara, A. Morandi, P. Chiarugi, S. Crotti, M. Agostini, M. Montopoli, I. Masgras, A. Rasola, R. Garcia-Escudero, N. Delaleu, A. Rinaldi, F. Bertoni, J. de Bono, A. Carracedo, A. Alimonti, Compartmentalized activities of the pyruvate dehydrogenase complex sustain lipogenesis in prostate cancer. Nat. Genet. 50, 219–228 (2018). 29335542 10.1038/s41588-017-0026-3 PMC5810912

[22] Y. Wang, Y. R. Guo, K. Liu, Z. Yin, R. Liu, Y. Xia, L. Tan, P. Yang, J. H. Lee, X. J. Li, D. Hawke, Y. Zheng, X. Qian, J. Lyu, J. He, D. Xing, Y. J. Tao, Z. Lu, KAT2A coupled with the α-KGDH complex acts as a histone H3 succinyltransferase. Nature 552, 273–277 (2017). 29211711 10.1038/nature25003 PMC5841452

[23] O. Yogev, O. Yogev, E. Singer, E. Shaulian, M. Goldberg, T. D. Fox, O. Pines, Fumarase: A mitochondrial metabolic enzyme and a cytosolic/nuclear component of the DNA damage response. PLoS Biol. 8, e1000328 (2010). 20231875 10.1371/journal.pbio.1000328 PMC2834712

[24] R. Nagaraj, R. Kim, A. T. Clark, F. Chi, U. Banerjee, M. S. Sharpley, Y. Zhou, D. Braas, Nuclear localization of mitochondrial TCA cycle enzymes as a critical step in mammalian zygotic genome activation. Cell 168, 210–223.e11 (2017). 28086092 10.1016/j.cell.2016.12.026 PMC5321559

[25] R. C. Sun, V. V. Dukhande, Z. Zhou, L. E. A. Young, S. Emanuelle, C. F. Brainson, M. S. Gentry, Nuclear glycogenolysis modulates histone acetylation in human non-small cell lung cancers. Cell Metab. 30, 903–916.e7 (2019). 31523006 10.1016/j.cmet.2019.08.014 PMC6834909

[26] R. J. DeBerardinis, A. Mancuso, E. Daikhin, I. Nissim, M. Yudkoff, S. Wehrli, C. B. Thompson, Beyond aerobic glycolysis: Transformed cells can engage in glutamine metabolism that exceeds the requirement for protein and nucleotide synthesis. Proc. Natl. Acad. Sci. U.S.A. 104, 19345–19350 (2007). 18032601 10.1073/pnas.0709747104 PMC2148292

[27] L. J. Reitzer, B. M. Wice, D. Kennell, Evidence that glutamine, not sugar, is the major energy source for cultured HeLa cells. J. Biol. Chem. 254, 2669–2676 (1979). 429309

[28] B. J. Altman, Z. E. Stine, C. V. Dang, From Krebs to clinic: Glutamine metabolism to cancer therapy. Nat. Rev. Cancer 16, 619–634 (2016). 27492215 10.1038/nrc.2016.71 PMC5484415

[29] K. E. Wellen, G. Hatzivassiliou, U. M. Sachdeva, T. V. Bui, J. R. Cross, C. B. Thompson, ATP-citrate lyase links cellular metabolism to histone acetylation. Science 324, 1076–1080 (2009). 19461003 10.1126/science.1164097 PMC2746744

[30] V. Iacobazzi, V. Infantino, Citrate—New functions for an old metabolite. Biol. Chem. 395, 387–399 (2014). 24445237 10.1515/hsz-2013-0271

[31] D. D. Clarke, Fluoroacetate and fluorocitrate: Mechanism of action. Neurochem. Res. 16, 1055–1058 (1991). 1784332 10.1007/BF00965850

[32] R. J. Molenaar, J. P. Maciejewski, J. W. Wilmink, C. J. F. van Noorden, Wild-type and mutated IDH1/2 enzymes and therapy responses. Oncogene 37, 1949–1960 (2018). 29367755 10.1038/s41388-017-0077-z PMC5895605

[33] U. C. Okoye-Okafor, B. Bartholdy, J. Cartier, E. N. Gao, B. Pietrak, A. R. Rendina, C. Rominger, C. Quinn, A. Smallwood, K. J. Wiggall, A. J. Reif, S. J. Schmidt, H. Qi, H. Zhao, G. Joberty, M. Faelth-Savitski, M. Bantscheff, G. Drewes, C. Duraiswami, P. Brady, A. Groy, S.-R. Narayanagari, I. Antony-Debre, K. Mitchell, H. R. Wang, Y.-R. Kao, M. Christopeit, L. Carvajal, L. Barreyro, E. Paietta, H. Makishima, B. Will, N. Concha, N. D. Adams, B. Schwartz, M. T. McCabe, J. Maciejewski, A. Verma, U. Steidl, New IDH1 mutant inhibitors for treatment of acute myeloid leukemia. Nat. Chem. Biol. 11, 878–886 (2015). 26436839 10.1038/nchembio.1930 PMC5155016

[34] J. Son, C. A. Lyssiotis, H. Ying, X. Wang, S. Hua, M. Ligorio, R. M. Perera, C. R. Ferrone, E. Mullarky, N. Shyh-Chang, Y. Kang, J. B. Fleming, N. Bardeesy, J. M. Asara, M. C. Haigis, R. A. DePinho, L. C. Cantley, A. C. Kimmelman, Glutamine supports pancreatic cancer growth through a KRAS-regulated metabolic pathway. Nature 496, 101–105 (2013). 23535601 10.1038/nature12040 PMC3656466

[35] M. Uhlén, L. Fagerberg, B. M. Hallström, C. Lindskog, P. Oksvold, A. Mardinoglu, Å. Sivertsson, C. Kampf, E. Sjöstedt, A. Asplund, I. Olsson, K. Edlund, E. Lundberg, S. Navani, C. A.-K. Szigyarto, J. Odeberg, D. Djureinovic, J. O. Takanen, S. Hober, T. Alm, P.-H. Edqvist, H. Berling, H. Tegel, J. Mulder, J. Rockberg, P. Nilsson, J. M. Schwenk, M. Hamsten, K. von Feilitzen, M. Forsberg, L. Persson, F. Johansson, M. Zwahlen, G. von Heijne, J. Nielsen, F. Pontén, Proteomics. Tissue-based map of the human proteome. Science 347, 1260419 (2015). 25613900 10.1126/science.1260419

[36] S. Agarwal, M. C. Sharma, P. Jha, P. Pathak, V. Suri, C. Sarkar, K. Chosdol, A. Suri, S. S. Kale, A. K. Mahapatra, P. Jha, Comparative study of IDH1 mutations in gliomas by immunohistochemistry and DNA sequencing. Neuro Oncol. 15, 718–726 (2013). 23486690 10.1093/neuonc/not015 PMC3661098

[37] P. De, R. Chatterjee, Evidence of nucleolar succinic dehydrogenase activity. Exp. Cell Res. 27, 172–173 (1962). 13884459 10.1016/0014-4827(62)90061-7

[38] M. Mackmull, M. Iskar, L. Parca, S. Singer, P. Bork, A. Ori, M. Beck, Histone deacetylase inhibitors (HDACi) cause the selective depletion of bromodomain containing proteins (BCPs). Mol. Cell Proteomics 14, 1350–1360 (2015). 25755299 10.1074/mcp.M114.042499 PMC4424404

[39] D. I. Kim, S. C. Jensen, K. A. Noble, B. Kc, K. H. Roux, K. Motamedchaboki, K. J. Roux, An improved smaller biotin ligase for BioID proximity labeling. Mol. Biol. Cell 27, 1188–1196 (2016). 26912792 10.1091/mbc.E15-12-0844 PMC4831873

[40] Y. Liao, A. Castello, B. Fischer, S. Leicht, S. Föehr, C. K. Frese, C. Ragan, S. Kurscheid, E. Pagler, H. Yang, J. Krijgsveld, M. W. Hentze, T. Preiss, The cardiomyocyte RNA-binding proteome: Links to intermediary metabolism and heart disease. Cell Rep. 16, 1456–1469 (2016). 27452465 10.1016/j.celrep.2016.06.084 PMC4977271

[41] R. H. Malty, H. Aoki, A. Kumar, S. Phanse, S. Amin, Q. Zhang, Z. Minic, F. Goebels, G. Musso, Z. Wu, H. Abou-tok, M. Meyer, V. Deineko, S. Kassir, V. Sidhu, M. Jessulat, N. E. Scott, X. Xiong, J. Vlasblom, B. Prasad, L. J. Foster, T. Alberio, B. Garavaglia, H. Yu, G. D. Bader, K. Nakamura, J. Parkinson, M. Babu, A map of human mitochondrial protein interactions linked to neurodegeneration reveals new mechanisms of redox homeostasis and NF-κB signaling. Cell Syst. 5, 564–577.e12 (2017). 29128334 10.1016/j.cels.2017.10.010 PMC5746455

[42] Y. Liu, Y. Mi, T. Mueller, S. Kreibich, E. G. Williams, A. Van Drogen, C. Borel, M. Frank, P. L. Germain, I. Bludau, M. Mehnert, M. Seifert, M. Emmenlauer, I. Sorg, F. Bezrukov, F. S. Bena, H. Zhou, C. Dehio, G. Testa, J. Saez-Rodriguez, S. E. Antonarakis, W. D. Hardt, R. Aebersold, Multi-omic measurements of heterogeneity in HeLa cells across laboratories. Nat. Biotechnol. 37, 314–322 (2019). 30778230 10.1038/s41587-019-0037-y

[43] B. T. Weinert, C. Schölz, S. A. Wagner, V. Iesmantavicius, D. Su, J. A. Daniel, C. Choudhary, Lysine succinylation is a frequently occurring modification in prokaryotes and eukaryotes and extensively overlaps with acetylation. Cell Rep. 4, 842–851 (2013). 23954790 10.1016/j.celrep.2013.07.024

[44] K. Y. Lee, A. Chopra, G. L. Burke, Z. Chen, J. F. Greenblatt, K. K. Biggar, M. D. Meneghini, A crucial RNA-binding lysine residue in the Nab3 RRM domain undergoes SET1 and SET3-responsive methylation. Nucleic Acids Res. 48, 2897–2911 (2020). 31960028 10.1093/nar/gkaa029 PMC7102954

[45] Y. Yang, G. E. Gibson, Succinylation links metabolism to protein functions. Neurochem. Res. 44, 2346–2359 (2019). 30903449 10.1007/s11064-019-02780-x PMC6755074

[46] C. Chinopoulos, The mystery of extramitochondrial proteins lysine succinylation. Int. J. Mol. Sci. 22, 6085 (2021). 34199982 10.3390/ijms22116085 PMC8200203

[47] A. Geladaki, N. K. Britovšek, L. M. Breckels, T. S. Smith, O. L. Vennard, C. M. Mulvey, O. M. Crook, L. Gatto, K. S. Lilley, Combining LOPIT with differential ultracentrifugation for high-resolution spatial proteomics. Nat. Commun. 10, 331 (2019). 30659192 10.1038/s41467-018-08191-w PMC6338729

[48] A. M. Intlekofer, L. W. S. Finley, Metabolic signatures of cancer cells and stem cells. Nat. Metab. 1, 177–188 (2019). 31245788 10.1038/s42255-019-0032-0 PMC6594714

[49] D. Xu, F. Shao, X. Bian, Y. Meng, T. Liang, Z. Lu, The evolving landscape of noncanonical functions of metabolic enzymes in cancer and other pathologies. Cell Metab. 33, 33–50 (2021). 33406403 10.1016/j.cmet.2020.12.015

[50] A. Ori, A. Andrés-Pons, M. Beck, The use of targeted proteomics to determine the stoichiometry of large macromolecular assemblies. Methods Cell Biol. 122, 117–146 (2014). 24857728 10.1016/B978-0-12-417160-2.00006-0

[51] H. H. Kanani, M. I. Klapa, Data correction strategy for metabolomics analysis using gas chromatography–mass spectrometry. Metab. Eng. 9, 39–51 (2007). 17052933 10.1016/j.ymben.2006.08.001

[52] C. Jungreuthmayer, S. Neubauer, T. Mairinger, J. Zanghellini, S. Hann, ICT: Isotope correction toolbox. Bioinformatics 32, 154–156 (2015). 26382193 10.1093/bioinformatics/btv514

[53] M. Kumar, M. Gouw, S. Michael, H. Sámano-Sánchez, R. Pancsa, J. Glavina, A. Diakogianni, J. A. Valverde, D. Bukirova, J. Signalyševa, N. Palopoli, N. E. Davey, L. B. Chemes, T. J. Gibson, ELM-The eukaryotic linear motif resource in 2020. Nucleic Acids Res. 48, D296–D306 (2020). 31680160 10.1093/nar/gkz1030 PMC7145657

[54] M. Seiler, A. Mehrle, A. Poustka, S. Wiemann, The 3of5 web application for complex and comprehensive pattern matching in protein sequences. BMC Bioinformatics 7, 1–12 (2006). 16542452 10.1186/1471-2105-7-144 PMC1523217

[55] K. J. Roux, D. I. Kim, M. Raida, B. Burke, A promiscuous biotin ligase fusion protein identifies proximal and interacting proteins in mammalian cells. J. Cell Biol. 196, 801–810 (2012). 22412018 10.1083/jcb.201112098 PMC3308701

[56] M. Mackmull, B. Klaus, I. Heinze, M. Chokkalingam, A. Beyer, R. B. Russell, A. Ori, M. Beck, Landscape of nuclear transport receptor cargo specificity. Mol. Syst. Biol. 13, 962 (2017). 29254951 10.15252/msb.20177608 PMC5740495

[57] I. Zemp, T. Wild, M. F. O’Donohue, F. Wandrey, B. Widmann, P. E. Gleizes, U. Kutay, Distinct cytoplasmic maturation steps of 40S ribosomal subunit precursors require hRio2. J. Cell Biol. 185, 1167–1180 (2009). 19564402 10.1083/jcb.200904048 PMC2712965

[58] M. Reichel, Y. Liao, M. Rettel, C. Ragan, M. Evers, A.-M. Alleaume, R. Horos, M. W. Hentze, T. Preiss, A. A. Millar, In planta determination of the mRNA-binding proteome of arabidopsis etiolated seedlings. Plant Cell 28, 2435–2452 (2016). 27729395 10.1105/tpc.16.00562 PMC5134986

[59] T. Strucko, K. Zirngibl, F. Pereira, E. Kafkia, E. T. Mohamed, M. Rettel, F. Stein, A. M. Feist, P. Jouhten, K. R. Patil, J. Forster, Laboratory evolution reveals regulatory and metabolic trade-offs of glycerol utilization in Saccharomyces cerevisiae. Metab. Eng. 47, 73–82 (2018). 29534903 10.1016/j.ymben.2018.03.006

[60] H. Franken, T. Mathieson, D. Childs, G. M. A. Sweetman, T. Werner, I. Tögel, C. Doce, S. Gade, M. Bantscheff, G. Drewes, F. B. M. Reinhard, W. Huber, M. M. Savitski, Thermal proteome profiling for unbiased identification of direct and indirect drug targets using multiplexed quantitative mass spectrometry. Nat. Protoc. 10, 1567–1593 (2015). 26379230 10.1038/nprot.2015.101

[61] M. E. Ritchie, B. Phipson, D. Wu, Y. Hu, C. W. Law, W. Shi, G. K. Smyth, *limma* powers differential expression analyses for RNA-sequencing and microarray studies. Nucleic Acids Res. 43, e47 (2015). 25605792 10.1093/nar/gkv007 PMC4402510

[62] W. Huber, A. von Heydebreck, H. Sültmann, A. Poustka, M. Vingron, Variance stabilization applied to microarray data calibration and to the quantification of differential expression. Bioinformatics 18, S96–S104 (2002). 12169536 10.1093/bioinformatics/18.suppl_1.s96

[63] L. Gatto, K. S. Lilley, MSnbase-an R/Bioconductor package for isobaric tagged mass spectrometry data visualization, processing and quantitation. Bioinformatics 28, 288–289 (2012). 22113085 10.1093/bioinformatics/btr645

[64] D. Mellacheruvu, Z. Wright, A. L. Couzens, J. P. Lambert, N. A. St-Denis, T. Li, Y. V. Miteva, S. Hauri, M. E. Sardiu, T. Y. Low, V. A. Halim, R. D. Bagshaw, N. C. Hubner, A. Al-Hakim, A. Bouchard, D. Faubert, D. Fermin, W. H. Dunham, M. Goudreault, Z. Y. Lin, B. G. Badillo, T. Pawson, D. Durocher, B. Coulombe, R. Aebersold, G. Superti-Furga, J. Colinge, A. J. R. Heck, H. Choi, M. Gstaiger, S. Mohammed, I. M. Cristea, K. L. Bennett, M. P. Washburn, B. Raught, R. M. Ewing, A. C. Gingras, A. I. Nesvizhskii, The CRAPome: A contaminant repository for affinity purification-mass spectrometry data. Nat. Methods 10, 730–736 (2013). 23921808 10.1038/nmeth.2557 PMC3773500

[65] S. R. Bhatnagar, Y. Yang, B. Khundrakpam, A. C. Evans, M. Blanchette, L. Bouchard, C. M. T. Greenwood, An analytic approach for interpretable predictive models in high-dimensional data in the presence of interactions with exposures. Genet. Epidemiol. 42, 233–249 (2018). 29423954 10.1002/gepi.22112 PMC6175336

[66] Z. Gu, R. Eils, M. Schlesner, Complex heatmaps reveal patterns and correlations in multidimensional genomic data. Bioinformatics 32, 2847–2849 (2016). 27207943 10.1093/bioinformatics/btw313

[67] J. Reimand, T. Arak, P. Adler, L. Kolberg, S. Reisberg, H. Peterson, J. Vilo, g:Profiler-A web server for functional interpretation of gene lists (2016 update). Nucleic Acids Res. 44, W83–W89 (2016). 27098042 10.1093/nar/gkw199 PMC4987867

[68] A. Bateman, UniProt: A worldwide hub of protein knowledge. Nucleic Acids Res. 47, D506–D515 (2019). 30395287 10.1093/nar/gky1049 PMC6323992

[69] J. D. R. Knight, H. Choi, G. D. Gupta, L. Pelletier, B. Raught, A. I. Nesvizhskii, A.-C. Gingras, ProHits-viz: A suite of web tools for visualizing interaction proteomics data. Nat. Methods 14, 645–646 (2017). 28661499 10.1038/nmeth.4330 PMC5831326

[70] I. Bergiers, T. Andrews, Ö. V. Bölükbaşı, A. Buness, E. Janosz, N. Lopez-Anguita, K. Ganter, K. Kosim, C. Celen, G. I. Perçin, P. Collier, B. Baying, V. Benes, M. Hemberg, C. Lancrin, Single-cell transcriptomics reveals a new dynamical function of transcription factors during embryonic hematopoiesis. eLife 7, 1–38 (2018). 10.7554/eLife.29312 PMC5860872 29555020

[71] J. Schindelin, I. Arganda-Carreras, E. Frise, V. Kaynig, M. Longair, T. Pietzsch, S. Preibisch, C. Rueden, S. Saalfeld, B. Schmid, J. Y. Tinevez, D. J. White, V. Hartenstein, K. Eliceiri, P. Tomancak, A. Cardona, Fiji: An open-source platform for biological-image analysis. Nat. Methods 9, 676–682 (2012). 22743772 10.1038/nmeth.2019 PMC3855844

[72] L. Gatto, S. Gibb, J. Rainer, MSnbase, efficient and elegant R-based processing and visualization of raw mass spectrometry data. J. Proteome Res. 20, 1063–1069 (2021). 32902283 10.1021/acs.jproteome.0c00313

[73] L. Gatto, L. M. Breckels, S. Wieczorek, T. Burger, K. S. Lilley, Mass-spectrometry-based spatial proteomics data analysis using pRoloc and pRolocdata. Bioinformatics 30, 1322–1324 (2014). 24413670 10.1093/bioinformatics/btu013 PMC3998135

[74] H. Wickham, M. Averick, J. Bryan, W. Chang, L. McGowan, R. François, G. Grolemund, A. Hayes, L. Henry, J. Hester, M. Kuhn, T. Pedersen, E. Miller, S. Bache, K. Müller, J. Ooms, D. Robinson, D. Seidel, V. Spinu, K. Takahashi, D. Vaughan, C. Wilke, K. Woo, H. Yutani, Welcome to the Tidyverse. J. Open Source Softw. 4, 1686 (2019).

[75] M. D. Young, M. J. Wakefield, G. K. Smyth, A. Oshlack, Gene ontology analysis for RNA-seq: Accounting for selection bias. Genome Biol. 11, R14 (2010). 20132535 10.1186/gb-2010-11-2-r14 PMC2872874

[76] T. Geiger, A. Wehner, C. Schaab, J. Cox, M. Mann, Comparative proteomic analysis of eleven common cell lines reveals ubiquitous but varying expression of most proteins. Mol. Cell. Proteomics 11, M111 (2012). 10.1074/mcp.M111.014050 PMC3316730 22278370

[77] Y. Benjamini, Y. Hochberg, Controlling the false discovery rate: A practical and powerful approach to multiple testing. J. R. Stat. Soc. Series B Stat. Methodol. 57, 289–300 (1995).

[78] Y. Perez-Riverol, A. Csordas, J. Bai, M. Bernal-Llinares, S. Hewapathirana, D. J. Kundu, A. Inuganti, J. Griss, G. Mayer, M. Eisenacher, E. Pérez, J. Uszkoreit, J. Pfeuffer, T. Sachsenberg, Ş. Yilmaz, S. Tiwary, J. Cox, E. Audain, M. Walzer, A. F. Jarnuczak, T. Ternent, A. Brazma, J. A. Vizcaíno, The PRIDE database and related tools and resources in 2019: Improving support for quantification data. Nucleic Acids Res. 47, D442–D450 (2019). 30395289 10.1093/nar/gky1106 PMC6323896

[79] K. Haug, K. Cochrane, V. C. Nainala, M. Williams, J. Chang, K. V. Jayaseelan, C. O’Donovan, MetaboLights: A resource evolving in response to the needs of its scientific community. Nucleic Acids Res. 48, D440–D444 (2020). 31691833 10.1093/nar/gkz1019 PMC7145518

