# Supplementary material for: Operation of a TCA cycle subnetwork in the mammalian nucleus
Source: Sci Adv. 2022 Aug 31;8(35):eabq5206. doi: 10.1126/sciadv.abq5206 (PMC9432838; doi:10.1126/sciadv.abq5206)
Supplement: Supplementary file 1 — Figs. S1 to S12 [file sciadv.abq5206_sm.pdf]

Science Advances
AAAS

Supplementary Materials for

Operation of a TCA cycle subnetwork in the mammalian nucleus

Eleni Kafkia *et al.*

Corresponding author: Kiran Raosaheb Patil, kp533@mrc-tox.cam.ac.uk

Sci. Adv. **8**, eabq5206 (2022)
DOI: 10.1126/sciadv.abq5206

The PDF file includes:

Figs. S1 to S12
Legends for tables S1 to S14

Other Supplementary Material for this manuscript includes the following:

Tables S1 to S14

Supplementary Figures

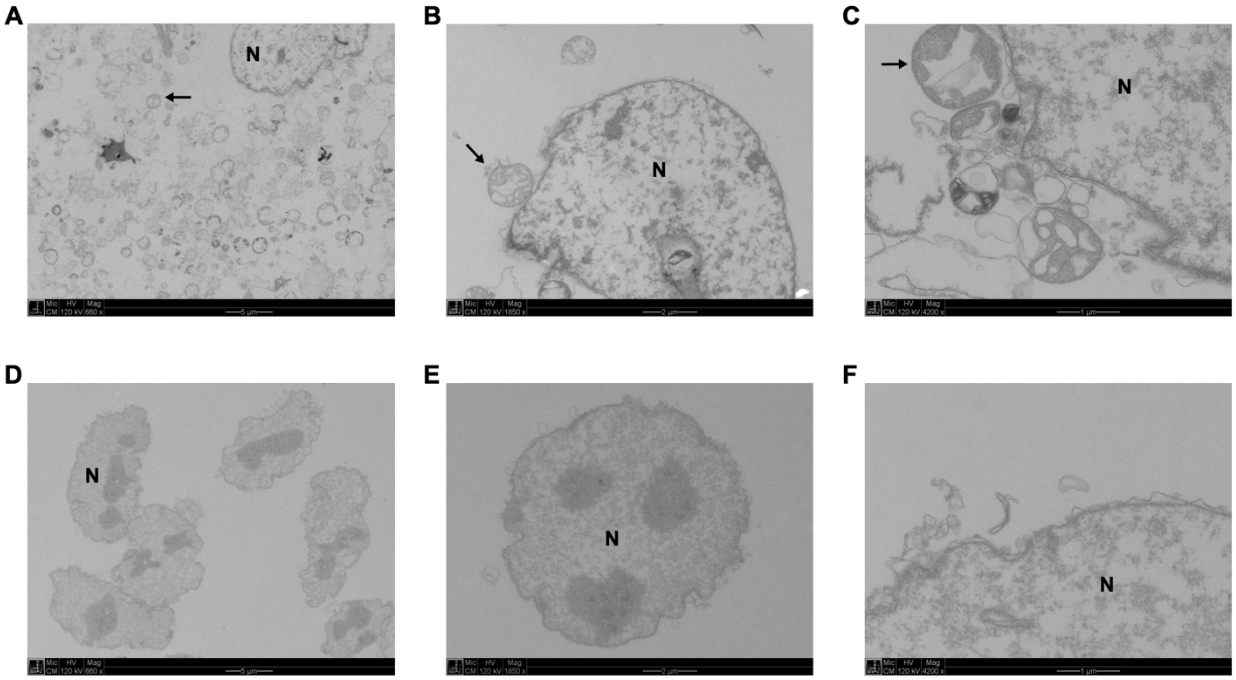

Fig. S1. Representative electron microscopy images from lysed cells (A to C) and isolated nuclei (D to F). For both lysed cells and isolated nuclei, the following magnifications are presented: x660 (A and D), x1850 (B and E) and x4200 (C and F). The letter “N” indicates a representative nucleus. The arrows point to mitochondria.

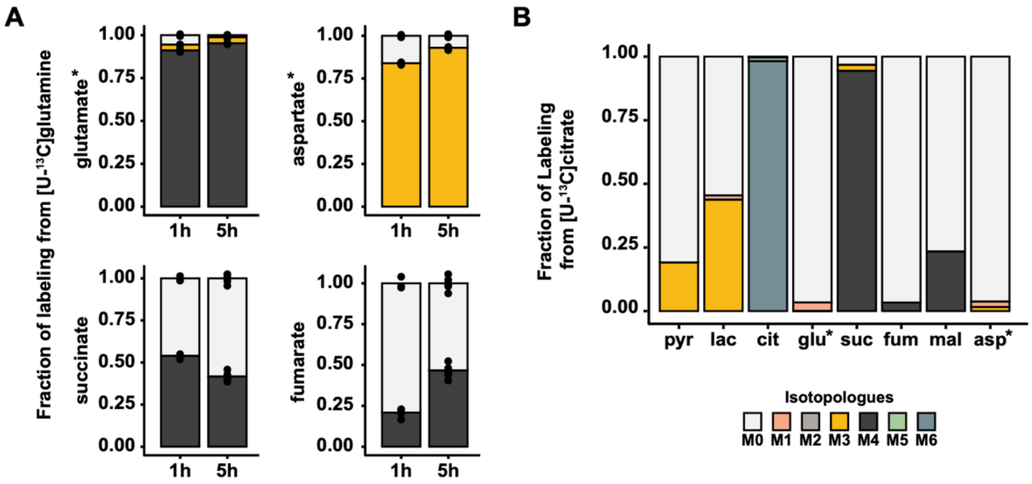

Fig. S2. (A) Fraction of labeling of the different mass isotopologues for glutamate, aspartate, succinate and fumarate in nuclei incubated for 1 (1h) and 5 hours (5h) with [U-13C]glutamine. For the mass isotopologues (Mn; n, number of 13C-carbons) legend refer to panel B. For the 1 hour incubation, n = 3 biological replicates. For the 5 hours incubation, n = 6 biological replicates. (B) Fraction of labeling of the different mass isotopologues (Mn; n, number of 13C-carbons) for each metabolite in lysed cells incubated with [U-13C]citrate for 5 hours. One representative experiment is depicted. In panels A and B, the quantified ions for glutamate and aspartate correspond to a four and a three-carbon fragment, respectively. Data are presented as mean of the indicated biological replicates with individual data points shown. Abbreviations: pyr, pyruvate; lac, lactate; cit, citrate; glu, glutamate; suc, succinate; fum, fumarate; mal, malate; asp, aspartate.

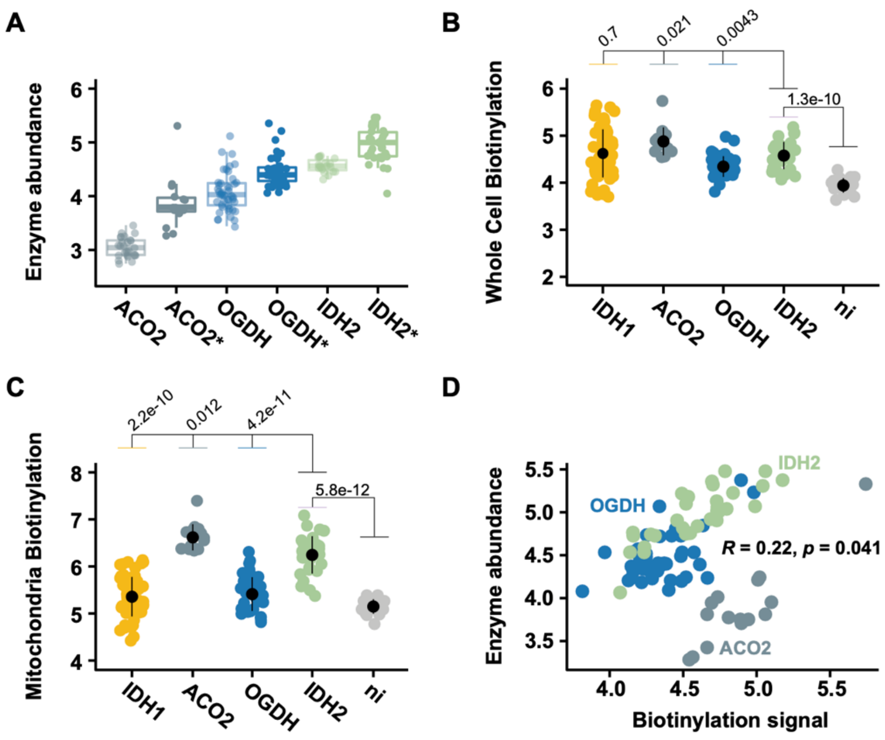

Fig S3. (A) Abundance levels of the bait enzymes before and after (demarcated by *) induction of their expression. The quantification was based on immunofluorescence microscopy using antibodies specific to the respective endogenous enzymes. From left to right, n = 27, 15, 45, 40, 19 and 31 cells quantified per case. (B and C) Quantification of the biotinylated proteins in the whole cell area (B) and in the mitochondria (C) of cells expressing the indicated bait enzymes. The assessment was based on immunofluorescence microscopy using fluorescently-labeled streptavidin. “ni” refers to a cell line where the expression of the bait enzyme and the biotinylation was not induced; for the particular case we used the cell line of OGDH. For panels B and C, from left to right, n = 60, 15, 40, 31 and 19 quantified cells. (D) Pearson correlation analysis of the enzyme abundance levels (defined in panel A) with the biotinylation levels (defined in panel B) in cells expressing the indicated bait enzymes. For ACO2, n = 15; for OGDH, n = 40; for IDH2, n = 31. In panels (A) to (D), points represent quantification (log2 scale) of

individual cells with the population mean and standard deviation calculated for each case. In panels (A) to (C), significance was assessed for the indicated comparisons using the Wilcoxon rank sum test.

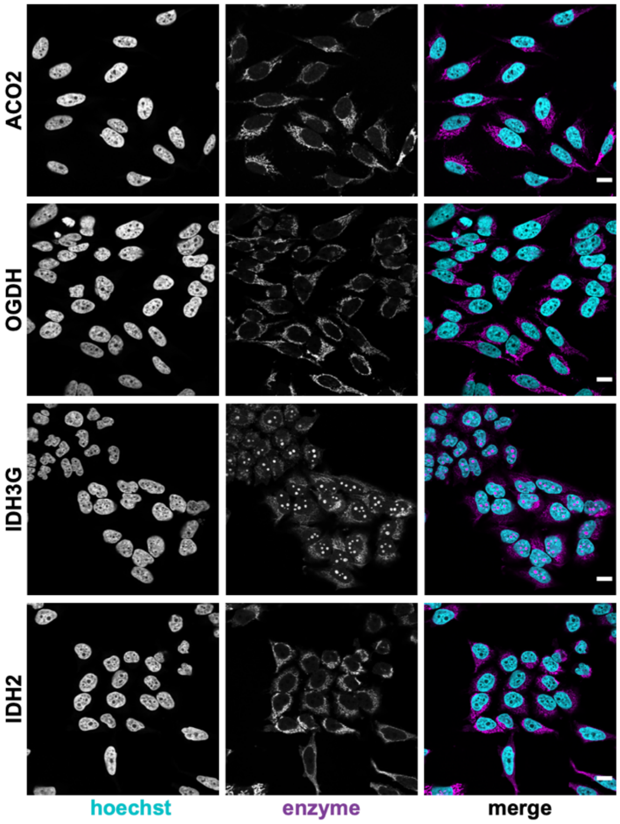

Fig S4. Subcellular distribution of the bait enzymes in *HeLa* cells. The localization was detected using antibodies specific to the endogenous enzymes (magenta). DNA was stained with Hoechst (cyan). Scale bars, 15μm.

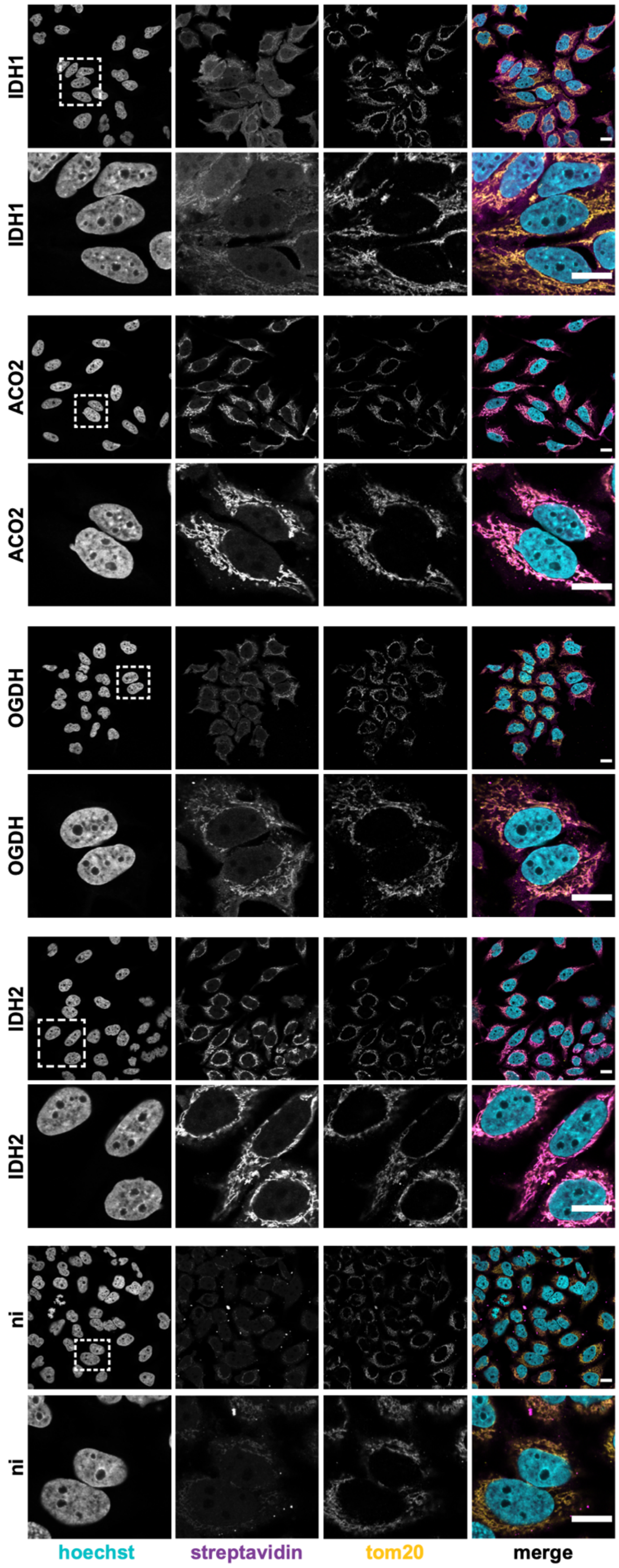
IDH1
IDH1
ACO2
ACO2
OGDH
OGDH
IDH2
IDH2
ni
ni
hoechst
streptavidin
tom20
merge

Fig S5. Subcellular distribution of the biotinylated proteins in the cell lines expressing the bait enzymes. The biotinylation was detected using fluorescently labeled streptavidin (magenta). Cells were also stained for the outer mitochondrial membrane protein Tom20 (yellow), and DNA (cyan). For each case, a wide-field (upper panel) and a close-up (lower panel) view are depicted. “ni” refers to cells where the expression of a bait enzyme and the biotinylation has not been induced. Scale bars, 15μm.

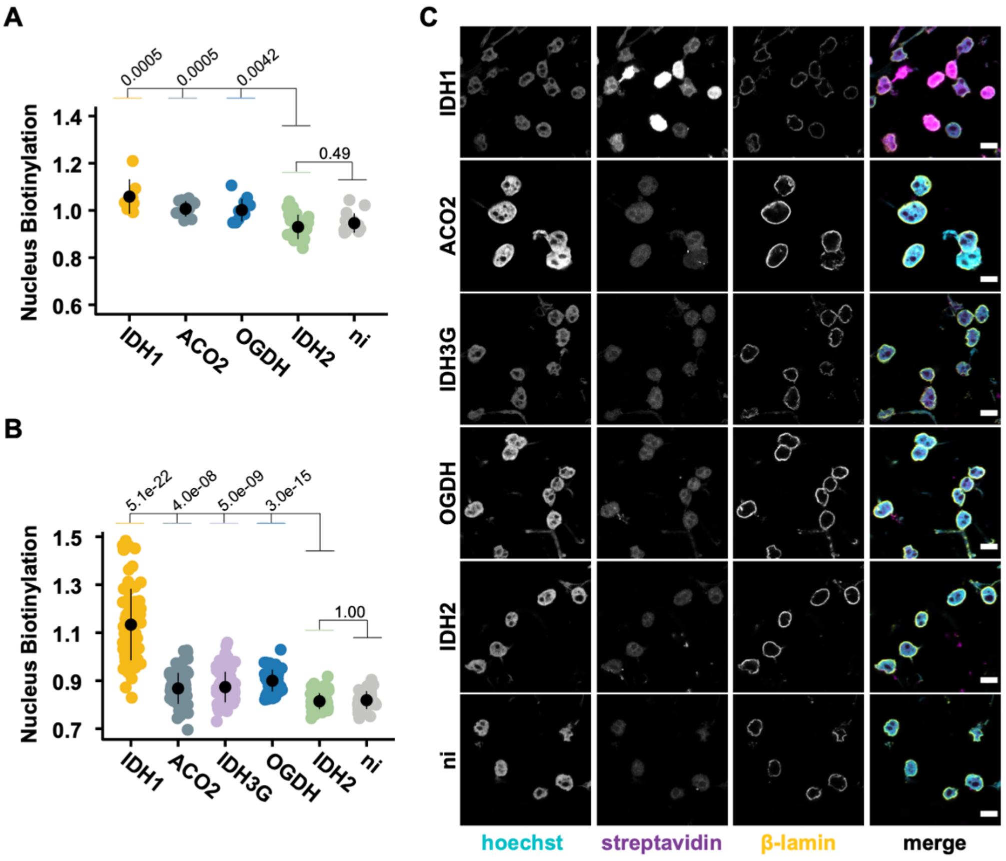

Fig S6. (A and B) Quantification of the biotinylated proteins in the nucleus region of lysed cells (A) and in isolated nuclei (B) from cells expressing the bait enzymes. The quantification was based on immunofluorescence microscopy using fluorescently labeled streptavidin. “ni” refers to cells where the expression of a bait enzyme and the biotinylation has not been induced. Points represent quantification (log_2 scale) of individual nuclei with the population mean and standard deviation calculated for each case. Significance was assessed for the indicated comparisons using the Wilcoxon rank sum test. For panel A, from left to right, n = 7, 12, 11, 24 and 15 quantified nuclei. For panel B, from left to right, n = 73, 75, 69, 55, 63 and 37 quantified nuclei. (C) Representative immunofluorescence images of nuclei isolated from cells expressing the bait enzymes. The nuclei were stained

for the biotinylated proteins with fluorescently labeled streptavidin (magenta), for the nuclear membrane protein β-lamin (yellow) and for DNA (cyan). Scale bars, 15μm.

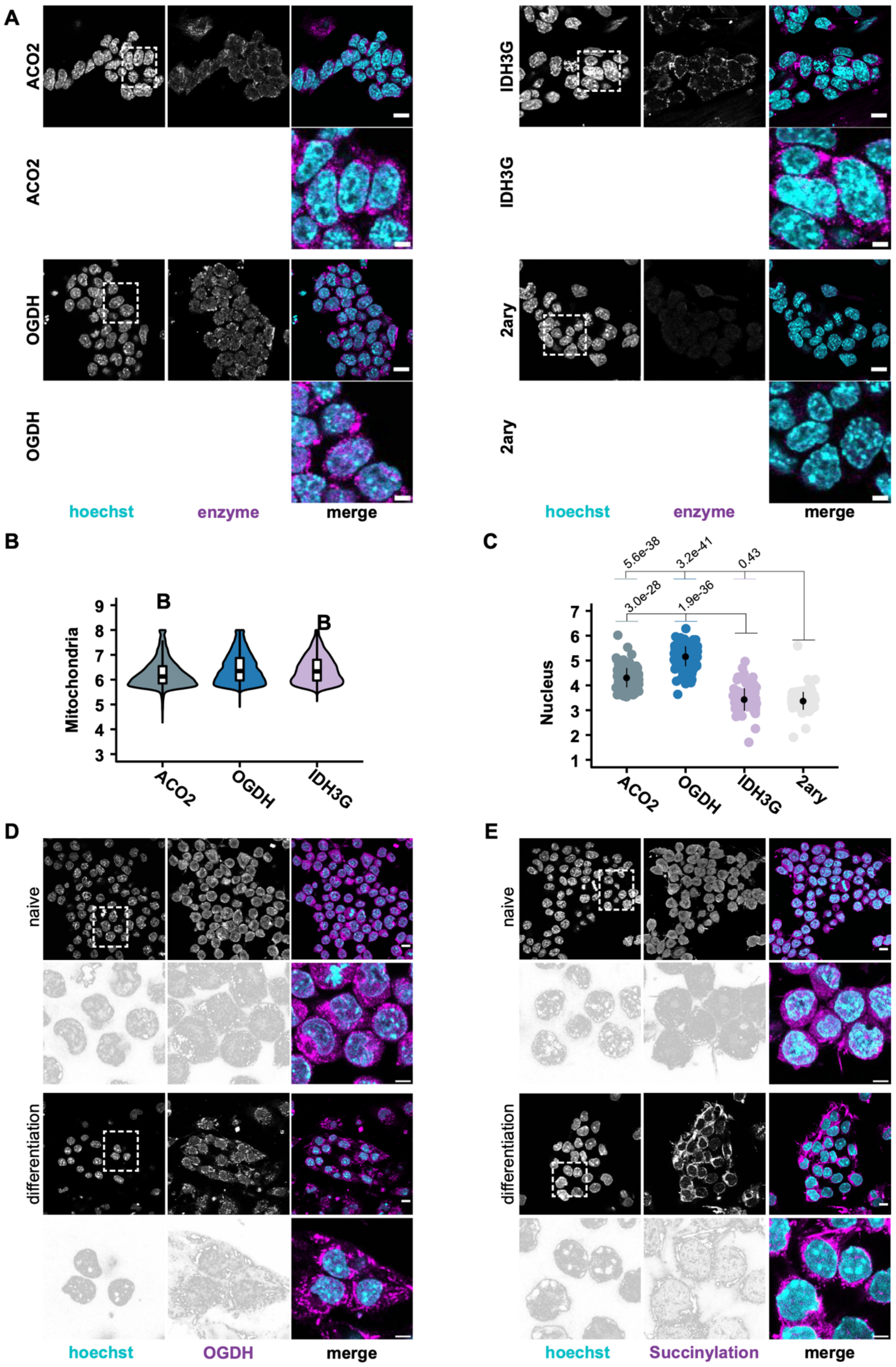
A
ACO2
ACO2
OGDH
OGDH
hoechst
enzyme
merge
IDH3G
IDH3G
2ary
2ary
hoechst
enzyme
merge
B
Mitochondria
B
B
ACO2
OGDH
IDH3G
C
5.6e-38
3.2e-41
0.43
3.0e-28
1.9e-36
Nucleus
ACO2
OGDH
IDH3G
2ary
D
naive
differentiation
hoechst
OGDH
merge
E
naive
differentiation
hoechst
Succinylation
merge

Fig S7. (A) Representative immunofluorescence images of embryonic stem cells stained for ACO2, OGDH and IDH3G. The detection was based on antibodies specific to the enzymes (magenta). DNA was stained with Hoechst (cyan). “2ary” refers to cells stained only with the secondary, fluorophore-conjugated, antibody. One representative plane is depicted. For each case, a wide-field (upper panel) and a close-up (lower panel) view are depicted. Scale bars for upper and lower panels are 15 μm and 5 μm, respectively. (B and C) Quantification of the enzymes levels in the mitochondria (B) and in the nuclei (C) of embryonic stem cells. In (C), points represent quantification (log_2 scale) of individual nuclei with the population mean and standard deviation calculated for each case. Significance was assessed for the indicated comparisons using the Wilcoxon rank sum test. For panel B, from left to right, n = 7467, 18457 and 2846 quantified mitochondria spots. For panel C, from left to right, n = 183, 166, 132 and 133 quantified nuclei. (D) Representative immunofluorescence images of OGDH in naive mouse embryonic stem cells and in differentiated counterparts. The detection was based on antibodies against OGDH (magenta). (E) Representative immunofluorescence images of succinylation in naive mouse embryonic stem cells and in differentiated counterparts. The detection was based on antibodies against succinyl lysine moieties (magenta). For (D and E), DNA was stained with Hoechst (cyan). In (D and E), a wide-field (upper panel) and a close-up (lower panel) view are depicted. Scale bars for upper and lower panels are 10 μm and 5 μm, respectively.

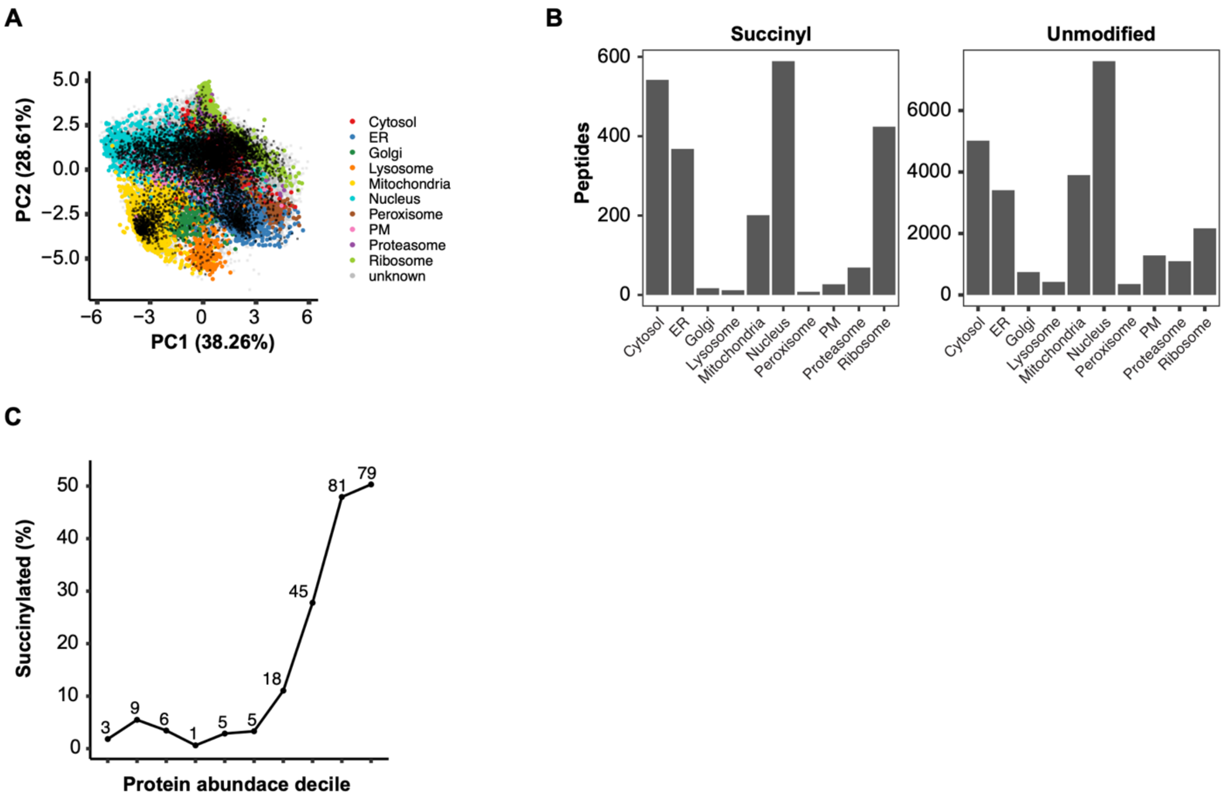

Fig S8. Localization of succinylated peptides by isotope tagging (LOPIT). (A) PCA projections of unmodified peptides, colored by their status as a priori markers of subcellular niches. Succinylated peptides identified by LOPIT are projected onto the same coordinates and shown as black dots. (B) Localization assignments for unmodified and succinylated peptides. Peptides that did not cross the threshold for assignment are excluded. (C) Percentage of proteins with at least one succinylated peptide, split into deciles of protein abundance, using (76). The number of succinylated proteins in each decile is indicated.

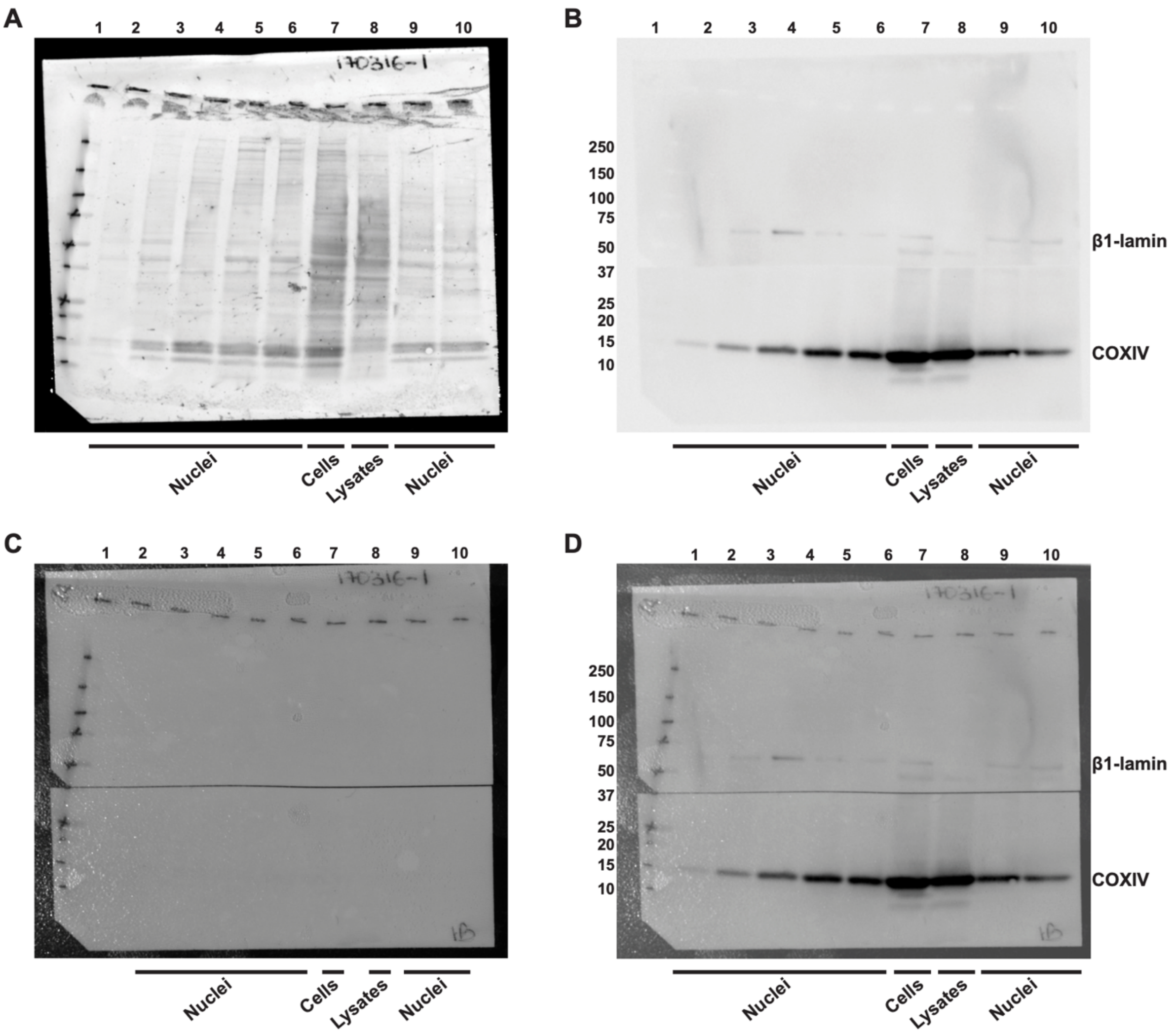

Fig S9. Immunoblot analysis for the inner mitochondrial membrane protein, COXIV, in isolated nuclei, cells and whole cell lysates (related to Fig. 1B). (A) Amido black staining of the membrane for detection of total proteins. (B) The membrane was cut in two pieces and the lower part was incubated with the antibody specific to COXIV. The upper part of the membrane was incubated with the antibody specific to β1-lamin. The chemiluminescent image of the membrane is displayed. The numbers on the left side of the panel correspond to the molecular weight (kD) of the protein standards. (C) Colorimetric image of the membrane. (D) Overlay of the chemiluminescent (panel B) and colorimetric (panel C) images. Well 1, protein standards (10 to 250 kD); wells 2 to 5,

isolated nuclei; well 6, cells; well 7, cell lysates; wells 8 to 9, isolated nuclei. The wells loaded with nuclei samples correspond to independent isolations.

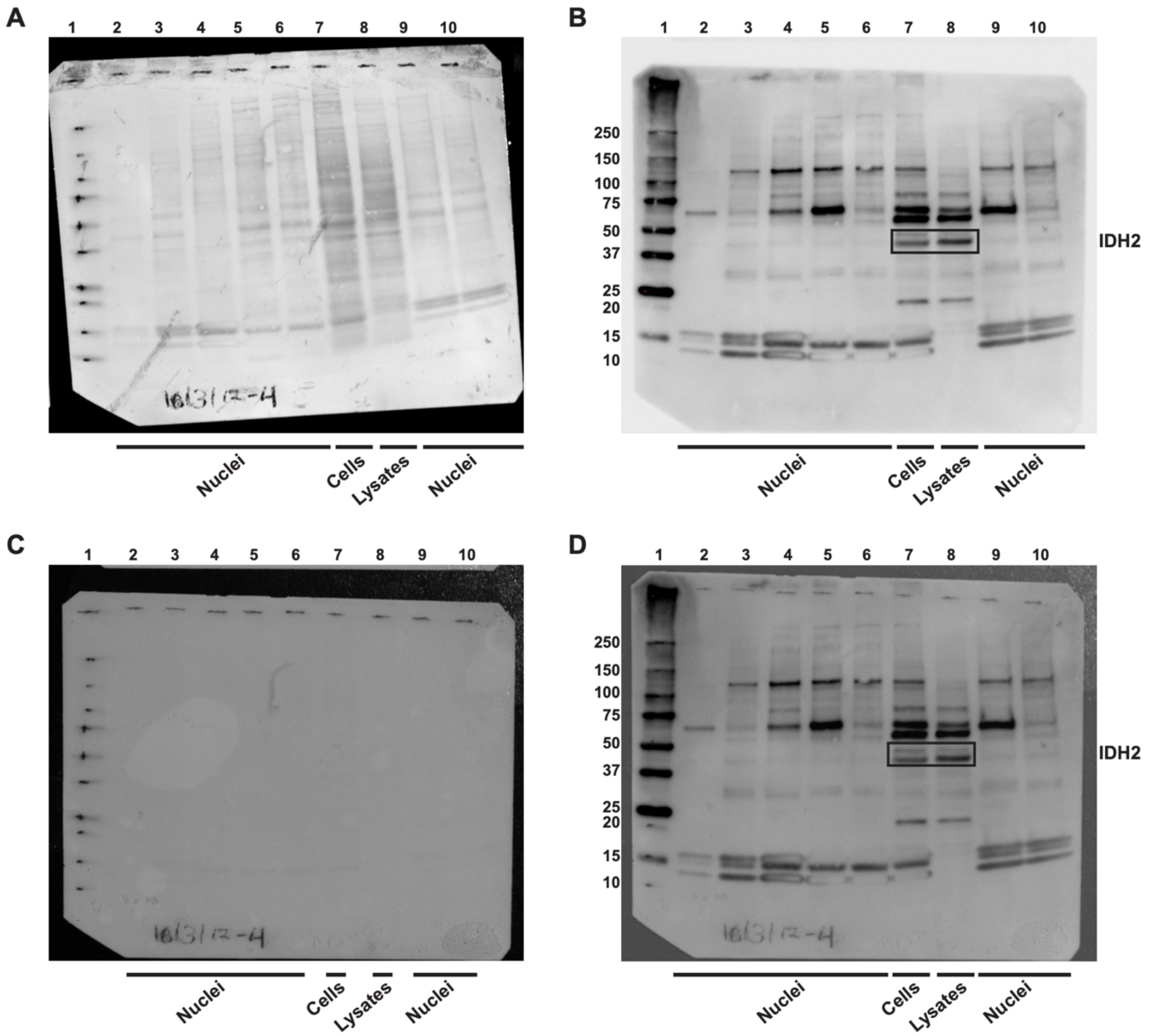

Fig S10. Immunoblot analysis for the mitochondrial matrix protein, IDH2, in isolated nuclei, cells and whole cell lysates (related to Fig. 1B). (A) Amido black staining of the membrane for detection of total proteins. (B) Chemiluminescent image of the membrane following incubation with antibody specific to IDH2. The band that corresponds to IDH2 according to the expected molecular weight of the enzyme are highlighted with a black box. The numbers on the left side of the panel correspond to the molecular weight (kD) of the protein standards. (C) Colorimetric image of the membrane. (D) Overlay of the chemiluminescent (panel B) and colorimetric (panel C) images. Well 1, protein standards

(10 to 250 kD); wells 2 to 5, isolated nuclei; well 6, cells; well 7, cell lysates; wells 8 to 9, isolated nuclei. The wells loaded with nuclei samples correspond to independent isolations.

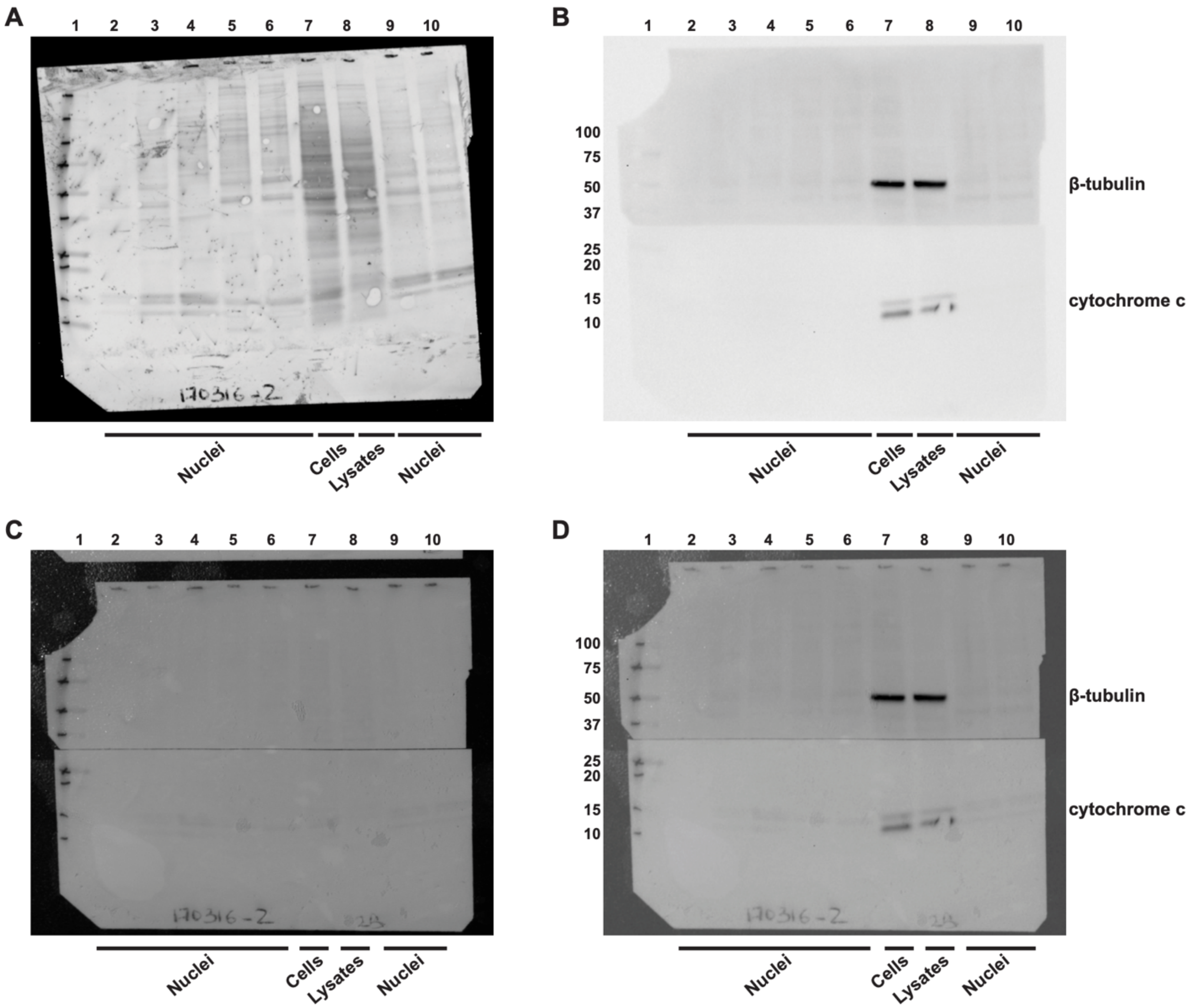

Fig S11. Immunoblot analysis of isolated nuclei, cells and whole cell lysates for the cytoplasmic protein β-tubulin and the mitochondrial shuttling protein cytochrome c (cyt c) (related to Fig. 1B). (A) Amido black staining of the membrane for detection of total proteins. (B) The membrane was cut in two pieces; the upper part was incubated with the antibody specific to β-tubulin and the lower part for cytochrome c. The numbers on the left side of the panel correspond to the molecular weight (kD) of the protein standards. (C) Colorimetric image of the membrane. (D) Overlay of the chemiluminescent (panel B) and colorimetric (panel C) images. Well 1, protein standards (10 to 250 kD); wells 2 to 5,

isolated nuclei; well 6, cells; well 7, cell lysates; wells 8 to 9, isolated nuclei. The wells loaded with nuclei samples correspond to independent isolations.

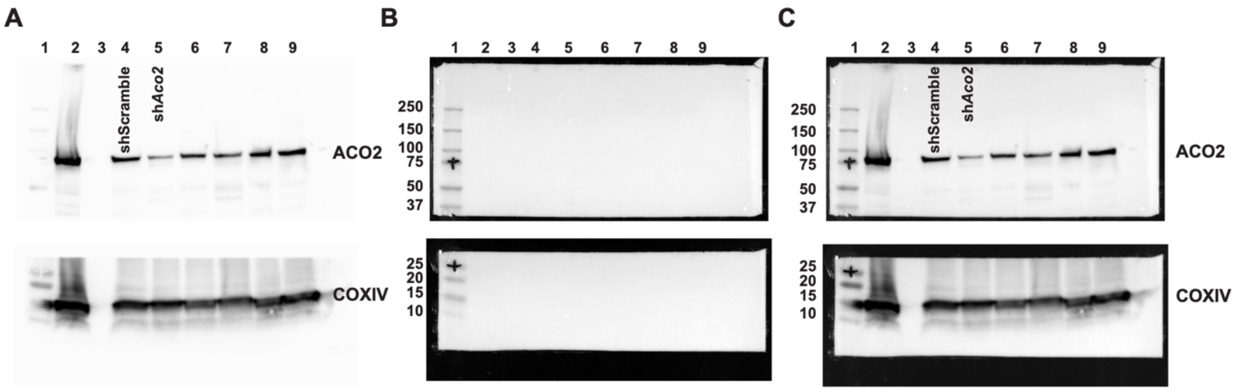

Fig S12. Immunoblot analysis for aconitase 2 (ACO2) in *HeLa* cells following shRNA mediated knockdown of ACO2. (A) The membrane was cut in two pieces; the upper part was incubated with an antibody specific to ACO2 and the lower part for COXIV. Well 2 corresponds to wild type *HeLa* cells, well 4 corresponds to a non-targeting shRNA control (shScramble), and wells 5 to 9 correspond to *HeLa* cells treated with five different shRNAs targeting ACO2. For the metabolomic experiments in isolated nuclei (related to Fig. 2G), the clone with the biggest reduction in ACO2 expression levels was utilized (well 5). Well 1 corresponds to a protein standard (10 to 250 kD). (B) Colorimetric image of the membrane. (C) Overlay of the chemiluminescent (panel A) and colorimetric (panel B) images. In panels (B) and (C), the numbers on the left side correspond to the molecular weight (kD) of the protein standard.

Legends for Tables S1 to S14

Table S1. Mass isotopologues of each metabolite in isolated nuclei incubated with [U-^{13}C]substrates. Data were acquired with GC-MS and were corrected for natural abundance isotopes. The different mass isotopologues per metabolite (column A) are indicated by "Mn" where n corresponds to the number of ^{13}C-carbons (see also Table S3).

Table S2. Mass isotopologues of each metabolite in whole cell lysates incubated with [U-^{13}C]substrates. Data were acquired with GC-MS and were corrected for natural abundance isotopes. The different mass isotopologues per metabolite (column A) are indicated by "Mn" where n corresponds to the number of ^{13}C-carbons (see also Table S3).

Table S3. Ion fragments for the quantification of the metabolites with GC-MS. Column B refers to the metabolite following the chemical derivatization with methoxyamine hydrochloride and *N*-methyl-*N*-(trimethylsilyl)trifluoroacetamide.

Table S4. Mass isotopologues of each metabolite in isolated nuclei and whole cell lysates incubated with [U-^{13}C]substrates. The different mass isotopologues per metabolite (column A) are indicated by "Mn" where n corresponds to the number of ^{13}C-carbons. Data were acquired with LC-MS and were corrected for natural abundance isotopes.

Table S5. Mass isotopologues of each metabolite in isolated nuclei derived from cells with shRNA-mediated knockdown of ACO2. Control samples correspond to isolated nuclei derived from cells treated with a non-specific shRNA (shScramble) Data were acquired with LC-MS and were not corrected for natural abundance isotopes. The

different mass isotopologues per metabolite (column A) are indicated by “Mn” where n corresponds to the number of ^{13}C-carbons.

Table S6. Mass isotopologues of each metabolite in isolated nuclei, whole cell lysates and isolated nuclear membranes. Data were acquired with LC-MS and were not corrected for natural abundance isotopes. The different mass isotopologues per metabolite (column A) are indicated by “Mn” where n corresponds to the number of ^{13}C-carbons.

Table S7. Overview of the putative interactors associated with the bait enzymes of TCA cycle. The table is related to Fig. 3A. Columns A to C correspond to different identifiers for a biotinylated protein; NCBI Entrez Gene (A), UniProt ID (B) and Protein Name as per UniProt (C). Column D specifies the comparison between a cell line expressing a bait enzyme and the parental *HeLa* cells. The cells expressing the bait enzyme are represented with the NCBI Entrez Gene symbol of the corresponding bait enzyme followed by an *in-house* ID which is given in parenthesis (e.g. cells expressing the PDHB fused with the biotin ligase and a FLAG tag are given as PDHB(C027)). Note that, N-IDH2(N544) refers to the case of IDH2 where the biotin ligase and the FLAG tag were fused at the N-terminus of the enzyme. Columns E to I report a number of summary statistics (limma analysis) for the biotinylated proteins (columns A to C) and the indicated comparisons (column D). Column E refers to the average (across all biological replicates) $\log_2$-fold change of the abundance of the biotinylated proteins in the cells expressing a bait enzyme relative to the parental *HeLa* cells. Column F corresponds to the average $\log_2$ abundance level of each biotinylated protein across all samples. Column G is the moderated *t*-statistic. Columns H and I show the associated *p* value and the false discovery rate (fdr) adjustment for *p* values using the Benjamini and Hochberg’s method,

respectively. Column J reports the bait enzyme(s) with which a biotinylated protein is significantly associated. Column K refers to Fig. 3A and specifies the cluster a biotinylated protein belongs to. Columns L and M report the number of MS runs and biological replicates, respectively, each biotinylated protein was detected. Column N shows the number of quantified unique peptide matches per biotinylated protein.

Table S8. Gene ontology enrichment analysis for the clusters of correlating putative interactors across all bait enzymes of TCA cycle. The table is related to Fig. 3A. Each cluster (column A) was analyzed with g:Profiler for gene ontology enrichment for the terms depicted in column C; Biological Process (GO:BP), Cellular Component (GO:CC) and Molecular Function (GO:MF). The columns are a direct output from g:Profiler.

Table S9. Subcellular localization of the top putative interactors of each bait enzyme of TCA cycle. The table is related to Fig. 3B. Column A shows the bait enzyme defined by the NCBI Entrez Gene symbol. Columns B to D show different identifiers for the biotinylated proteins that were significantly enriched for each bait enzyme; NCBI Entrez Gene (B), UniProt ID (C) and Protein Name as per UniProt (D). Columns E to G were retrieved from UniProt database; Column E reports the UniProt's manual assignment of subcellular localization for the reviewed human genes corresponding to the respective biotinylated proteins. Columns H to I report the subcellular localization for each biotinylated protein as retrieved from Gene Ontology. Columns J to V refer to localization evidence from Human Protein Atlas. Column W provides the merged localization data from all previous databases. Column X reports the final localization category assigned to each biotinylated protein based on the information provided in Column W. “Nucleus” and “Mitochondria” refer to proteins only detected in the nucleus and mitochondria,

respectively. “Nucleus shared” refers to proteins that are detected in the nucleus and any other compartment. This category includes also the proteins that are detected in the nucleus and mitochondria. “Mitochondria shared” refers to proteins that are detected in the mitochondria and any other compartment excluding the nucleus. “Other” includes the proteins that are detected in any compartment other than the nucleus or the mitochondria. This column was utilized for the generation of Fig. 3B

Table S10. Destination vectors and entry clones for the generation of the engineered bait enzymes fused with the biotin ligase and a FLAG tag.

Table S11. Primers for the generation of the engineered constructs and oligos.

Table S12. Sequencing primers for the engineered constructs of the bait enzymes fused with the biotin ligase and a FLAG tag.

Table S13. Gene ontology enrichment analysis of the putative interactors of IDH2 carrying the biotin ligase at the N-terminus. The columns are a direct output from g:Profiler which was used for the gene ontology enrichment for the terms noted in column C; “Biological Process” (GO: BP), “Molecular Function” (GO: MF) and “Cellular Component” (GO: CC).

Table S14. Proteomics analysis of whole cell lysates, isolated nuclei and isolated nuclear membranes.
